# AI-based secure event-driven serverless architecture for scalable digital civic participation platform

**DOI:** 10.3389/frai.2026.1826465

**Published:** 2026-07-24

**Authors:** Aizhan Kassymova, Abdul Razaque, Raissa Uskenbayeva, Zhuldyz Kalpeyeva, Aizhan Anartayeva

**Affiliations:** 1 Department of Software Engineering, Satbayev University, Almaty, Kazakhstan; 2 Department of Cybersecurity, Satbayev University, Almaty, Kazakhstan; 3 School of Digital Public Administration, Astana IT University, Astana, Kazakhstan

**Keywords:** artificial intelligence, biometric verification, data localization, digital civic participation, digital sovereignty, event-driven architecture, serverless computing, smart city

## Abstract

**Introduction:**

With the growing digitalization of urban governance and the increasing demand for transparency, sustainability and secure decision-making, the need for scalable and intelligent digital civic platforms has been raised. However, current e-participation systems are often plagued by challenges related to scalability, regulatory compliance, digital sovereignty and secure citizen authentication. The challenges are tackled in this paper by proposing an AI-enabled serverless architecture for next generation digital civic engagement.

**Methods:**

This study proposes an AI-based Secure Event-driven Serverless Participation Architecture (SESPA) for digital e-participation services based on the Citizen Participation Event Model (CPEM), where each citizen interaction is treated as an event within a continuous decision-making process. Architecture employs an event-driven serverless computing paradigm integrated with artificial intelligence modules for biometric citizen verification and anomaly detection. To satisfy the digital sovereignty requirements of the Republic of Kazakhstan, a hybrid data localization model is introduced that separates personally identifiable information from anonymized analytical events. The proposed dual-loop architecture stores sensitive citizen data within national infrastructure while enabling cloud-based processing of anonymized event streams for scalable analytics without violating regulatory requirements.

**Results:**

An experimental prototype was evaluated under workloads of up to 10,000 concurrent users. The results demonstrated stable latency across p50, p75, p95, and p99 percentile metrics, efficient scalability through provisioned concurrency, and reduced total cost of ownership compared with an equivalent Kubernetes-based deployment. Moreover, the addition of AI modules to the event-processing pipeline added little latency overhead and allowed for precise detection of anomalous and suspicious participation behavior in controlled experimental workloads.

**Discussion and conclusion:**

The proposed SESPA architecture effectively combines event-driven serverless computing, AI-assisted security mechanisms and hybrid data localization to provide a secure, scalable and regulation-compliant digital participation platform. The results demonstrate that the proposed framework offers a good technology foundation for next generation smart city applications by supporting high citizen engagement, regulatory compliance, digital sovereignty and intelligent decision-making, while maintaining high system performance and cost efficiency.

## 1 Introduction

Urbanization, demographic expansion, and the digitization of public services complicate urban governance. Governments must promptly fulfill regulations, render unambiguous decisions, and engage all stakeholders in policymaking. In big cities, town hall meetings, public hearings, and paper petitions don't work very effectively to get people to talk to each other since not many people turn up, they cost a lot to run, and they can't be utilized by a lot of people at once. Governments all over the world are looking into digital platforms that let citizens use online tools and services to help make choices and administer the government (Rossello N. B. et al., 2025; Lartey et al., 2025). Decidim, Consul, and CitizenLab simplify city governance online. These platforms enable comments, online consultations, public project voting, and policy discussions. Many studies show that these systems make government institutions more transparent, responsible, and trustworthy (Dedema and Hagen, 2025; De Jaeger and Swerts, 2025). Despite their popularity, most modern e-participation platforms are monolithic or conventional client-server designs that need resource allocation and centralized computer infrastructure administration (Razaque et al., 2021). They struggle to handle sudden user activity surges, especially during elections or huge consultations, limiting performance and scalability (Calavaro et al., 2025). Recent advancements in cloud computing, big data infrastructures, and distributed computing paradigms present opportunities to reassess the architectural foundations of digital civic engagement platforms. With event-driven computing, computers can see human activities as independent events that launch asynchronous processing pipelines. This approach lets a lot of interactions happen in real time, scales intelligently as workloads vary, and is more fault-tolerant (Maciá-Lillo et al., 2025). In an event-driven design, everything from enrolling people to submitting proposals to voting to commenting may be seen as streaming events that scalable cloud services can manage. This helps people make decisions and analyze data almost right away. Serverless computing makes this idea even better by allowing apps to run functions when they need them without needing to keep the infrastructure running all the time. Serverless models cut costs, get rid of unnecessary resources, and help systems with a lot of traffic grow and shrink as needed (Jonas et al., 2019; Eivy, 2017). Smart cities have used serverless architectures quite well for processing large amounts of data, analyzing IoT data, and providing real-time urban services (Zhang et al., 2024). But they haven't been employed much yet in platforms for digital democracy and citizen participation. Artificial intelligence and machine learning are also being utilized more and more in public administration systems to help make choices and automate government procedures. AI methods can do more complicated things, such as discovering strange patterns, looking at behavior, and working out how individuals feel about feedback from citizens (Anthony and Sarshar, 2025; Bozkurt et al., 2025). Natural language processing (NLP) tools help governments look at a lot of feedback and suggestions from citizens, and computer vision methods can support experimental biometric verification workflows and identity validation mechanisms on safe participation platforms (Goodfellow et al., 2016; Ribeiro et al., 2016). When coupled with event-driven infrastructures, these technologies can provide smart participation settings that can alter based on how people respond and uncover possible fraud or manipulation in participatory processes. Another key facet of digital governance is following the standards for national data protection and digital sovereignty. Many governments require that data about their citizens be stored within their own borders or processed in very strict legal ways. Hybrid cloud designs that combine local data storage with public cloud services that can grow are a good solution to handle this challenge (Katal et al., 2013). Governments may preserve control of private data and use the power and scalability of cloud platforms with these kinds of solutions. Even with these new technologies, most research does not put these pieces together into one system for digital involvement. A lot of research only focuses on one area of the problem, like utilizing AI to look at what people say, establishing rules for smart-city data, or using serverless computing for massive apps. Not many full architectural models include all of these things at once: event-driven computing, serverless cloud architecture, AI-based analytics, biometric authentication, and hybrid data localization. So, it is clear that we need to do more research on how to build digital participation infrastructures that are safe, can handle heavy democratic processes, and are scalable, transparent, resilient, and legal. This work proposes an improved event-driven architecture for digital citizen participation services, based on a serverless cloud computing model, to address this shortcoming. The proposed architecture incorporates intelligent modules for biometric identification, behavioral analytics, real-time data processing, and anomaly detection. These modules will assist in keeping civic participation platforms safe and flexible. The research introduces a Citizen Participation Event Model (CPEM), which organizes user interactions as streaming events within a real-time decision-support framework. This method makes it easy to quickly handle participation activities like voting, registration, submitting proposals, and discussion. It can also handle a lot of work and still develop. The objectives of this study are summarized as:

- To provide a formal event-driven architectural model for platforms that let individuals take part in civic life online.
- To add AI parts, such as finding anomalies, checking for liveness, and using NLP to look at citizen feedback, to a streaming participation framework.
- To check how well the proposed serverless architecture works with real-world workloads, how well it scales, how reliable it is, and how cheap it is to run.
- To test how well the serverless model works compared to a Kubernetes deployment that employs containers.

The proposed SESPA framework was implemented as a functional cloud-based prototype using Amazon Web Services (AWS) serverless infrastructure components, including AWS Lambda, EventBridge, API Gateway, DynamoDB, Kinesis, and Rekognition. The experimental evaluation was conducted in a controlled cloud test environment using synthetic and workload-driven civic participation events designed to emulate realistic large-scale citizen interaction scenarios. Therefore, the present study should be interpreted as an empirically evaluated prototype implementation and architectural validation study, rather than a fully deployed national-scale production system or purely conceptual framework.

The proposed method aims to create a new generation of smart-city participation systems by combining cloud-native architectures with smart analytics and digital identification systems. These technologies will be able to change based on how citizens act, find unexpected behavior patterns, and make sure they are always available during big civic events. The results give us useful information that can help us build scalable digital democracy systems and improve how public government works in new smart-city ecosystems. The remainder of the article is organized as follows: Section 2 discusses the most relevant methods. Section 3 presents a mathematical model for the proposed event-driven civic participation platforms. Section 4 explains the proposed solution. Section 5 provides the results. Section 6 discusses the results, including limitations. Section 7 concludes the entire article.

### 1.1 Novelty and contributions

#### 1.1.1 Novelty of work

Digital civic participation systems use traditional web architectures and centralized processing methods, which make it difficult to scale, secure, and provide real-time decision assistance. Several solutions are introduced to address this issue, and most solutions focus on single pieces of technology, like blockchain voting systems, cloud-based participation portals, or standalone analytics tools. But these systems usually work on their own and do not offer a unified architecture that can handle large-scale citizen involvement with strong identity verification, real-time analytics, and compliance with regulatory data. The novelty of this research lies in creating a single scalable framework for digital civic participation platforms that combines artificial intelligence, biometric identity verification, streaming analytics, and hybrid data localization into one event-driven serverless architecture. The proposed design treats the citizens as a separate event that is processed by distributed serverless components. This method makes it possible to handle data in real time, scale systems up and down as needed, and change how systems respond to events. Another novelty is the inclusion of biometric identification verification and AI-based anomaly detection methods into the civic engagement process. This makes digital participation more reliable because it avoids identity theft and automated bot interactions. The architecture also includes a hybrid data localization mechanism that meets national data sovereignty standards and allows for large-scale cloud analytics. These features make the proposed architecture the next generation of scalable and secure digital governance platforms.

#### 1.1.2 Contributions of work

The main contributions are summarized as follows:

- The artificial intelligence mechanism is employed for abnormal participation detection behavior and fraudulent activities within civic platforms. Machine learning-based AVFDHD algorithm is used for securing biometric verification, AI-based fraud detection, spoofing detection, and privacy-preserving data processing to maintain a robust intelligent authentication system for digital platforms.
- This algorithm enables a secure, scalable, and event-driven civic participation system that authenticates users, validates participation actions, moderates the content, stores participation records, and generates real-time analytics for governance platforms. The event-driven civic participation workflow algorithm is used to analyze participation patterns, device characteristics, and behavioral features to identify suspicious interactions. The system integrity is improved by the integration of an anomaly, preventing automated manipulation and duplicate participation attempts.
- The challenge of identity verification in online civic engagement is addressed using facial recognition and liveness detection techniques. This approach is intended to support identity validation mechanisms within controlled experimental participation environments rather than an automated bot or impersonated identity.
- The study introduces a hybrid data localization model that separates sensitive personal data from anonymized analytical data. Personal information is securely stored within the national infrastructure to comply with digital sovereignty regulations. The anonymized participation data are processed in cloud environments for large-scale analytics and decision support. This dual-domain architecture enables strict compliance with national data protection requirements.

## 2 Related work

This section discusses the state-of-the-art approaches. A decentralized, AI-powered decision-supporting architecture for smart city government was put forth by Ning (2024). Using citizen data streams, machine learning algorithms in this architecture assist policymakers in making decisions immediately. To better meet the demands of the public, the framework processes massive volumes of urban data and employs predictive analytics. The best thing about this system is to handle a lot of citizen data and helps the government plan for the future. But the design is largely for decision-support analytics and does not contain the real-time citizen interaction pipelines, identity verification methods, or event-driven infrastructure that scalable e-participation systems need. Kim and Park (2024) conducted a study that introduced a blockchain-enabled digital participation platform designed to improve transparency and trust in public decision-making processes. The system uses distributed ledger technology to keep track of citizens for voting outcomes in a way that cannot be manipulated. The advantage of this approach is to prevent anyone from changing the records of participants. But solutions based on blockchain usually have trouble with scalability and high processing costs. Also, the proposed system does not have any adaptive AI modules for figuring out how people behave or finding strange things that happen throughout participation procedures. Garcia et al. (2024) designed a microservices-based architecture for smart city service platforms to enhance the scalability of public digital services. The approach divides municipal services into smaller microservices. The architecture indicates that city services may work together more easily and are more adaptable. Microservice-based systems still need their infrastructure to be kept up and their scaling processes to be done by hand, which can make them more expensive to run than serverless models. The proposed system also lacks streaming analytics capabilities essential for managing high-frequency citizen engagement events. Singh et al. (2024) proposed a cloud-based analytics framework for urban participation data obtained from mobile applications and digital consultation platforms, as big data gains significance in government. They use distributed cloud storage and machine learning algorithms to discover patterns and requests for city services. This strategy searches for large data sets and leverages interactions with citizens to come up with policy recommendations. But the technology generally works in batch mode, which makes it less appropriate for real-time participation services that demand immediate answers and help make decisions. Lopez et al. (2025) proposed a biometric authentication system for secure digital government services in the domain of digital identity verification. To make sure that someone is who they say they are before they may use government websites, the technology uses facial recognition and liveness detection algorithms. This framework is helpful since it is incredibly safe and can help keep people from stealing our identity when we communicate with them online. The research mainly focuses on authentication infrastructure and neglects to integrate biometric verification into a holistic participation framework that includes streaming analytics and citizen engagement services. Chen et al. (2024) proposed an edge-cloud collaborative architecture for real-time urban services to address performance issues in smart city applications. Their solution divides processing tasks between edge devices and cloud infrastructure, which makes it possible for apps that demand low latency to work well. In urban IoT contexts, the design makes it possible for systems to respond considerably faster and grow much more easily. The study is largely about smart city services that employ sensors. It doesn't really talk about how to integrate with digital democracy platforms or how to get citizens involved in decision-making. Kumar et al. (2024) also provided a useful contribution by building a framework for public policy feedback platforms that use AI to analyze sentiment. Their approach employs natural language processing to automatically categorize citizen comments and determine public sentiment regarding government initiatives. This approach is effective due to its capacity to manage a substantial volume of written remarks and aid administrators in understanding public preferences. It lacks a mechanism for managing significant events or real-time participation. Martinez et al. (2025) proposed the serverless architecture for scalable digital government applications that employ cloud-hosted function-as-a-service platforms. The solution dynamically allocates computing resources in accordance with incoming requirements, thereby reducing the time and cost associated with infrastructure administration. The most advantageous aspect of this architecture is its ability to manage workloads that are difficult to anticipate while simultaneously preserving minimal operating costs. The research concentrates on the routine provision of e-government services, except specialized modules for digital involvement, such as AI-driven anomaly detection in civic engagement systems, citizen identification verification, or voting procedures. The evaluated publications illustrate significant developments in the integration of blockchain, artificial intelligence, cloud computing, and biometric technology into digital governance systems. However, the majority of prior research has examined these components in isolation rather than as part of a unified infrastructure for engagement. At present, there are no digital democracy systems that simultaneously employ hybrid data localization, serverless execution, event-driven computing, biometric verification, and AI-based behavior analysis. In order to ensure that data is secure, accessible, and in accordance with national data standards, we continue to require a scalable, secure infrastructure that can accommodate numerous civic engagement projects. Table 1 illustrates a comparative examination of digital governance and AI-enhanced e-participation frameworks.

**Table 1:** Comparative analysis of digital governance and AI-enabled e-participation architectures.

Methods	Study focus	core approach	Evaluation method	AI/ML utilization	Application context	Personalization level	Key strengths	Limitations
Ning (2024)	AI decision support for smart city governance	AI-driven decision analytics framework	Experimental evaluation	Machine learning analytics	Smart city governance systems	Medium	Processes large citizen data streams and supports policy decision-making	Does not include real-time citizen interaction pipelines or identity verification
Kim and Park (2024)	Transparent digital participation	Blockchain-based voting and participation platform	Conceptual architecture validation	None (Blockchain focus)	Digital governance systems	Low–Medium	Immutable participation records and strong transparency	High computational cost and scalability challenges; lacks adaptive AI modules
Garcia et al. (2024)	Smart city service scalability	Microservices-based service architecture	Architectural evaluation	None	Urban digital service platforms	Low–Medium	Modular architecture improves flexibility and interoperability	Requires infrastructure management and lacks streaming analytics
Singh et al. (2024)	Citizen participation data analytics	Cloud-based big data analytics platform	Data analytics experiments	Machine learning analytics	Urban consultation and mobile participation platforms	Medium	Efficient analysis of large datasets and policy insights	Batch processing limits real-time citizen engagement
Lopez et al. (2025)	Secure digital identity verification	Biometric authentication using facial recognition and liveness detection	Security testing	Computer vision biometrics	Digital government platforms	Low	High security and fraud prevention	Focused only on authentication; lacks integration with engagement workflows
Chen et al. (2024)	Real-time urban services	Edge–cloud collaborative architecture	Performance evaluation	Distributed analytics	Smart city IoT applications	Low	Low latency and scalable processing for urban services	Designed mainly for sensor networks; limited integration with citizen participation
Kumar et al. (2024)	AI-assisted policy feedback analysis	NLP-based sentiment analysis framework	Analytical evaluation	Natural Language Processing	Public consultation platforms	Medium	Handles large volumes of citizen comments effectively	No support for real-time participation or event-driven architecture
Martinez et al. (2025)	Scalable e-government architecture	Serverless computing using FaaS	Cloud performance evaluation	Limited ML	Digital government service delivery	Low	Elastic scalability and reduced infrastructure cost	Does not integrate AI anomaly detection, identity verification, or voting modules
Proposed approach	Intelligent digital civic participation infrastructure	Event-driven serverless architecture with AI modules and hybrid data localization	Empirical performance testing with up to 10,000 concurrent users	AI-based anomaly detection, biometric verification, NLP analytics, streaming analytics	Smart city citizen participation platforms	High	Prototype-level demonstration of scalable participation processing, adaptive analytics, fraud detection, reduced TCO under evaluated cloud conditions (78–86%), and compliance with digital sovereignty requirements	Dependence on cloud platform limits and AI model quality; requires reliable network infrastructure

## 3 Mathematical model for proposed event-driven civic participation platforms

The proposed SESPA system is denoted by S, the set of citizens by U, government policymakers by G, the central civic participation platform by M, the AI and biometric security module by A, cloud infrastructure by Cl, civic participation events by E, external civic platforms by X, policy inputs by Y, and system outputs by O. The symbol *P* is avoided because it may be confused with platform, policymakers, policy inputs, or proposals. Thus, the proposed system is denoted by Equation 1:


$$\begin{array}{l} S=\left\{U,G,A,M,Cl,E,X,Y,O\right\} \end{array}$$ (1)

Each citizen *c*_*i*_∈ U generates one or more civic participation event sets *E*_*i*_, which are defined in Equation 2:


$$\begin{array}{l} E_{i}=\left\{e_{k}|e_{k}=\left(c_{i},t_{k},\tau_{k},d_{k}\right)\right\} \end{array}$$ (2)

where each event *e*_*k*_ is represented by Equation 3


$$\begin{array}{l} e_{k}=\left(c_{i},t_{k},\tau_{k},d_{k}\right) \end{array}$$ (3)

Where, *t*_*k*_ denotes the timestamp, τ_*k*_∈{*reg, vote, comment, proposal*} denotes event type, and *d*_*k*_ is the event data payload.

Thus, the total citizen-generated event stream *E*_*c*_ can be modeled in Equation 4:


$$\begin{array}{l} E_{c}=\overset{|U|}{\underset{i=1}{⋃}}E_{i} \end{array}$$ (4)

where *E*_*i*_ = {*e*_*i, k*_∣*e*_*i, k*_ = (*c*_*i*_, *t*_*i, k*_, τ_*i, k*_, *d*_*i, k*_), *k* = 1, …, ∣*E*_*i*_∣ }.

Prior to the processing of any event, it must undergo validation by the artificial intelligence *V*_*A*_ and biometric security module. Thus, the identity verification function can be defined in Equation 5:


$$\begin{array}{l} V_{A}:U\times ℰ\to \{0,1\}\\ V_{A}\left(c_{i},e_{k}\right)=\{\begin{array}{cc} 1, & \left(c_{i},e_{k}\right)\in V\\ 0, & \left(c_{i},e_{k}\right)\notin V \end{array} \end{array}$$ (5)

where *V*_*A*_(*c*_*i*_, *e*_*k*_) = 1 indicates that the citizen-event pair, *V*_*A*_(*c*_*i*_, *e*_*k*_) = 0 indicates failed verification, and V denotes the set of verified citizen-event pairs.

Only verified events are admitted into the platform. Hence, the validated event set E^*^ is determined in Equation 6:


$$\begin{array}{l} E^{*}=\left\{e_{k}\in E_{c}|V_{A}\left(c_{i},e_{k}\right)=1\right\} \end{array}$$ (6)

This guarantees the exclusion of fraudulent, duplicate, or unapproved events from subsequent processing. The central platform processes verified events through a serverless event orchestration function. Equation 7 defines the event orchestration/processing function of the SESPA platform.


$$\begin{array}{l} M:E^{*}\to O \end{array}$$ (7)

$$\begin{array}{l} M\left(e_{k}\right)=o_{k},\forall e_{k}\in E^{*} \end{array}$$ (8)

Equation 8 is defining how the orchestration function behaves for each verified event.

Where M denotes the central event-driven SESPA processing platform, and O denotes the set of processed outputs, and *o*_*k*_ denotes the processed output generated from the validated event *e*_*k*_.

Then Equation 9 should naturally continue as:


$$\begin{array}{l} o_{k}=M\left(c_{i},t_{k},\tau_{k},d_{k}\right),o_{k}\in O,\forall e_{k}\in E^{*} \end{array}$$ (9)

In Equation 10, the overall processed output stream is obtained as follows:


$$\begin{array}{l} O=\left\{o_{k}|o_{k}=M\left(e_{k}\right),\;e_{k}\in E^{*},\;k=1,\ldots ,m\right\} \end{array}$$ (10)

Since the platform is event-driven, the event load Λ(*t*) at time *t* can be modeled in Equation 11:


$$\begin{array}{l} \Lambda \left(t\right)=\overset{|E^{*}|}{\underset{k=1}{\sum }}1\left(t_{k}=t\right) \end{array}$$ (11)

where **1** is the indicator function. The serverless resource allocation can then be expressed in Equation 12:


$$\begin{array}{l} \text{Ψ}\left(t\right)=\alpha \Lambda \left(t\right),t\in \left[0,T\right] \end{array}$$ (12)

This demonstrates that the number of computational resources increases in direct proportion to the presence of new civic events.

where Ψ(*t*) denotes serverless resource allocation at time *t*, and α is the resource scaling coefficient.

Let the external civic participation platforms be represented by Equation 13


$$\begin{array}{l} X=\left\{x_{j}|1\leq j\leq q\right\} \end{array}$$ (13)

where *x*_*j*_ denotes platforms such as CitizenLab, Decidim, or Consul, and *q* denotes the total number of external platforms. The data records imported from external platform is provided in Equation 14.


$$\begin{array}{l} D_{X}=\left\{d_{j,k}\;|\;x_{j}\in X,\;1\leq j\leq q,\;1\leq k\leq |D_{x_{j}}|\right\}=\overset{q}{\underset{j=1}{⋃}}\overset{|D_{x_{j}}|}{\underset{k=1}{⋃}}d_{j,k} \end{array}$$ (14)

where *D*_*x*_*j*__denotes the data records imported from the external platform *x*_*j*_, and *d*_*j, k*_denotes the *k*-th data record from the platform *x*_*j*_.

The total participation data set used by the system given by Equation 15.


$$\begin{array}{l} D_{T}=D_{C}\cup D_{X} \end{array}$$ (15)

where *D*_*T*_ denotes the total participation dataset, *D*_*C*_ denotes the internally generated citizen participation dataset, and *D*_*X*_ denotes the imported external civic participation dataset.

Government policy makers submit policy inputs to the system. Each policy input can be modeled as follows by Equation 16:


$$\begin{array}{l} Y=\left\{y_{l}|1\leq l\leq r\right\} \end{array}$$

$$\begin{array}{l} y_{l}=\left(g_{l},\theta_{l},\omega_{l}\right)\in G\times \Theta \times \Omega ,l=1,2,\ldots ,r \end{array}$$ (16)

Where *y*_*l*_ denotes the *l*-th policy input, *g*_*l*_∈Gd denotes the responsible government policymaker, θ_*l*_∈Θ denotes the policy topic, ω_*l*_∈Ω denotes the policy objective parameter, and *r* denotes the total number of policy inputs.

The system combines citizen events and policy inputs through an aggregation function that can be determined by Equation 17:


$$\begin{array}{l} \Phi :D_{T}\times Y\to R_{A} \end{array}$$

OR


$$\begin{array}{l} R_{A}=\Phi \left(D_{T},Y\right) \end{array}$$ (17)

where Φ denotes the analytics aggregation function, *D*_*T*_ denotes the total participation dataset, and R_*A*_ denotes the generated analytics reports.

The analytics engine utilizes civic engagement data from the platform to compute participation and policy measures. It examines authenticated votes, proposals, comments, and registrations to uncover patterns of public interaction. Complex analytical techniques combine and evaluate inputs to ascertain patterns in policy initiatives, involvement, and public sentiment. Government stakeholders obtain organized reports and evidence-based policy recommendations from the indicators, facilitating data-driven decision-making. Let *R*_*A*_ = {*r*_1_, *r*_2_, …, *r*_*s*_} be the set of analytics reports. A general report function is written in Equation 18.


$$\begin{array}{l} r_{j}=\Phi \left(D_{T},Y\right),r_{j}\in R_{A},j=1,2,\ldots ,s \end{array}$$ (18)

Where *r*_*j*_ denotes the *j*-th analytics report, and *s* denotes the total number of generated reports.

A participation score *PS*_*j*_ for a given policy campaign should be defined in Equation 19.


$$\begin{array}{l} PS_{j}=w_{1}N_{vote,j}+w_{2}N_{prop,j}+w_{3}N_{comm,j}+w_{4}N_{reg,j} \end{array}$$ (19)

where *N*_*vote, j*_ denotes the number of validated votes, *N*_*prop, j*_ denotes the number of submitted proposals, *N*_*comm, j*_ denotes the number of citizen comments, *N*_*reg, j*_ is the number of completed registrations, and *w*_1_, *w*_2_, *w*_3_, *w*_4_ denote weighting coefficients.

The participation score of Equation 19 is a function of several indicators of civic engagement, the relative importance of which may vary according to the policy goals and participation priorities. Thus, the weighting coefficients have to satisfy normalization and proportionality constraints in order to obtain a balanced and mathematically correct participation scoring model. The feasible weight vector for the participation scoring model is presented in Equation 20. It guarantees that the weights of votes, proposals, comments and registrations are normalized, each weight is between 0 and 1 and all weights sum to 1. These constraints mean that the participation score is a valid weighted sum of the four indicators of civic engagement.


$$\begin{array}{l} W=\left\{\text{w}\in {\mathbb{R}}^{4}\;|\;\overset{4}{\underset{i=1}{\sum }}w_{i}=1,\;0\leq w_{i}\leq 1\right\} \end{array}$$ (20)

where ℝ^4^denotes the four-dimensional real-valued vector space associated with the weighting coefficients (*w*_1_, *w*_2_, *w*_3_, *w*_4_), corresponding to votes, proposals, comments, and registrations, respectively.

This weighted model can help quantify the level of civic engagement.

The system splits the data it collects into two groups to follow data sovereignty laws: sensitive personal data *D*_*P*_ and personal data *D*_*A*_ for analysis that has been made anonymous. Information about people that is not public includes documentation, which comprises biometric data, user identification documents, and registration credentials. The government needs to keep an eye on the infrastructure that stores these documents. But data that has been made anonymous for analysis of statistics on voting, proposal metrics, and engagement metrics without real names. By isolating distinct types of data, the framework makes it easier to follow national data protection rules. It also allows us to use cloud-based analytics and dynamic policy evaluations to our benefit. Equation 21 defines the data partitioning model of the proposed framework.


$$\begin{array}{lll} D_{P},D_{A} & \subseteq & D_{T} \end{array}$$

$$\begin{array}{l} \;D_{T}=D_{P}\cup D_{A},D_{P}\cap D_{A}=\emptyset \end{array}$$ (21)

where *D*_*P*_ denotes sensitive personal participation data, and *D*_*A*_ is anonymized analytical participation data.

A localization function that maps personal data to national storage and analytical data to cloud infrastructure is given in Equation 22.


$$\begin{array}{l} L:D_{T}\to \left\{N,C\right\}\\ \;L(d)=\{\begin{array}{cc} N, & d\in D_{P}\\ C, & d\in D_{A} \end{array} \end{array}$$ (22)

where *L* denotes the data-localization function, and Ndenotes sovereign national infrastructure.

The final system output consists of citizen notifications and government analytics reports that can be determined in Equation 23.


$$\begin{array}{l} S_{out}=\Gamma \left(E^{*},Y,D_{X}\right)=R_{C}\cup R_{A} \end{array}$$ (23)

where S_*out*_ denotes the final system output set, $R_{C}={\left\{r_{c_{i}}\right\}}_{i=1}^{|U|}$denotes citizen notification outputs, and Γ denotes the final response-generation function.

Therefore, the complete proposed architecture is expressed in compact form which can be determined in Equation 24.


$$\begin{array}{l} S_{out}=\Gamma \left(M\left(E^{*}\right),Y,D_{X}\;\right)\\ \;E^{*}\to M\left(E^{*}\right)\to O\to \Gamma \left(O,Y,D_{X}\right)\to S_{out} \end{array}$$

$$\begin{array}{l} S_{out}=\Gamma \left(O,Y,D_{X}\right)=R_{C}\cup R_{A} \end{array}$$ (24)

The architecture processes only authenticated civic events through the event-driven serverless platform, integrates government policy inputs and external civic platform data, and generates analytics reports and citizen notifications.

## 4 Proposed event-driven civic participation framework

**Figure 1:**
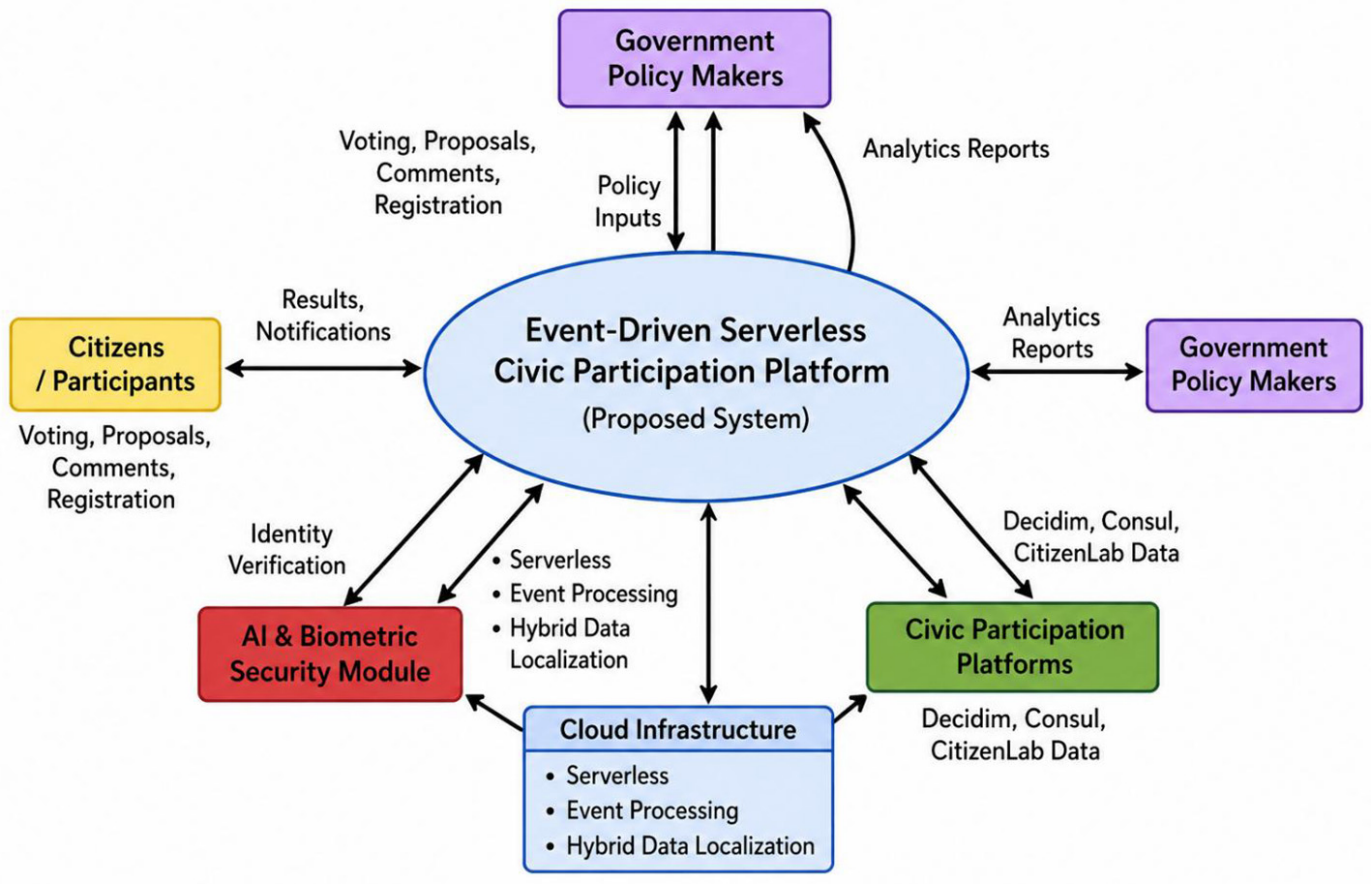
Conceptual architecture of the proposed SESPA integrating AI-based biometric verification, hybrid data localization, and cloud analytics for digital governance.

An event-driven serverless architecture was employed to construct and empirically test an intelligent digital civic engagement platform. The study's methodology was devised as a multi-stage, organized technique. The methodology encompasses the integration of artificial intelligence, architectural design, hybrid data localization modeling, and extensive experimental assessment. The architecture presented was implemented as a working prototype in a controlled AWS cloud environment for experimental verification and scalability evaluation, but not as a real governmental deployment. Figure 1 shows an idea of the proposed framework for civic participation that is based on events and does not require a server. The proposed architecture has a structure for civic participation that is based on events and does not use servers. This framework is meant to help with digital governance that is safe, open, and can grow. The framework uses modern cloud computing models, AI-based identity verification, and outside civic participation platforms to make it easier for citizens and government officials to work together in a structured way. Architecture is meant to fix some of the biggest problems with digital governance systems, such as how to make them bigger, how to keep data safe, how to check citizens' identities, and how to get feedback on policies. The event-driven serverless civic participation platform is the most important part of the proposed architecture. This is the most important layer that runs and organizes the system. This part handles everything that people do, such as giving feedback, asking questions about policies, collecting data, and looking at it. We do not have to manage the infrastructure separately because the platform uses serverless computing. The platform can also change size based on how many people are using it. An event-driven architecture lets the system quickly and asynchronously handle events with a lot of people. This lets it handle things that people do right away, like voting, leaving comments, or sending in proposals. The citizen interaction layer is the best way to get into the system. People and citizens can use the platform to sign up, vote, suggest new rules, and leave comments that everyone can see. The central platform takes these inputs, organizes them into events, and then looks at them to help people decide what to do. Then, the platform sends people the alerts and results. This maintains a transparent process and lets individuals give feedback all the time. People who work for the government can talk to the system using the Government Policy Makers interface. This helps them decide what to do and how to act. People in charge can utilize the platform to submit new regulations, topics for discussion, or policy ideas. Technology keeps track of how many people took part and utilizes their replies to provide suggestions for regulations and reports. These reports tell us how many people are involved, how public opinion is changing, and how well the plans are going. This helps governments make choices based on facts. The framework has a biometric security module that uses AI to identify us. This allows people to be honest and trust each other when they are online. This module uses AI and biometric identification to make sure users are who they say they are before they can accomplish anything. The security feature makes it tougher for anyone to register more than once, steal someone else's identity, or get into systems without permission. This makes it safer to vote and talk to people online.

The architecture also connects civic engagement platforms that are not part of the organization, such as CitizenLab, Decidim, and Consul. These platforms give people more tools and information so they can get involved. We can provide civic participation data from these sites to the main system. This shows us how many people are utilizing different platforms and helps us get more people to discuss policy. The cloud infrastructure layer is the foundation of the overall system. It lets us add more storage and processing power. There are pipes for managing events, contexts for running code without servers, and ways to maintain data in more than one place in this layer. Storing private data on a country's infrastructure is a way to keep information safe through hybrid data localization. It also lets the cloud analyze anonymous analytical data at the same time. This architecture makes sure that the rules of national data sovereignty are followed, while also making it easier for cloud-based analytics and growth. The proposed architecture creates a safe digital governance environment that can grow and is open to everyone. It also helps to make regulations that are based on data, gets more people involved, and makes the discussion bigger. Algorithm 1 illustrates the event-driven civic participation workflow.

Algorithm 1 **Event-driven civic participation processing workflow.**

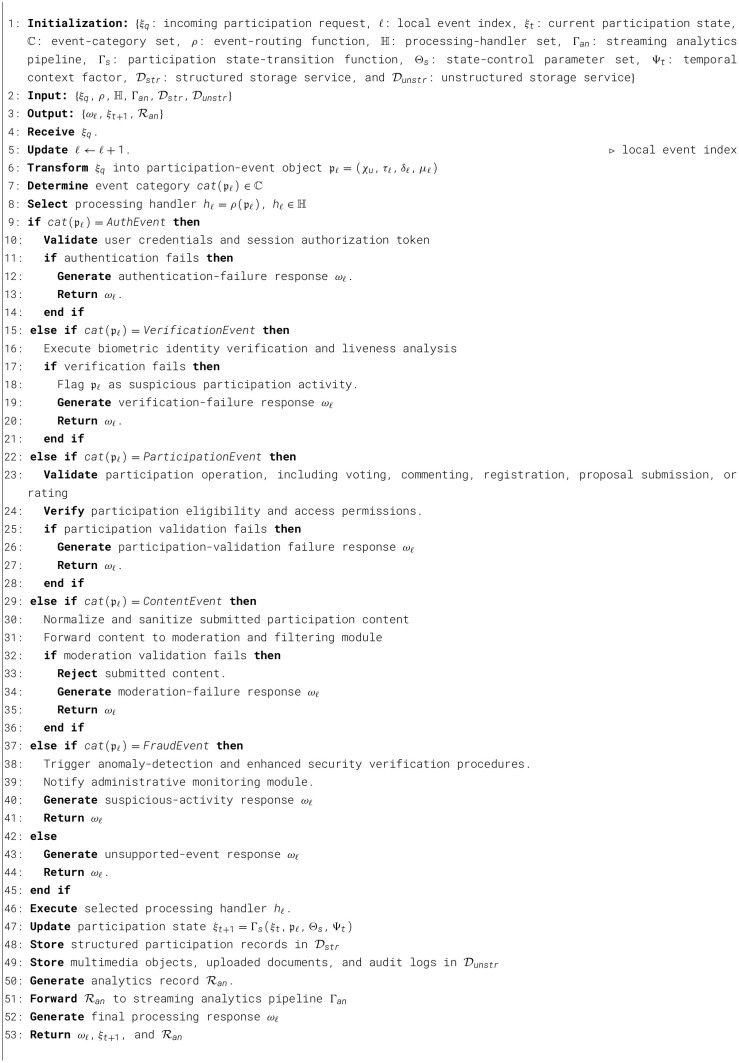

In Step 1, the algorithm initializes required system elements for event-driven civic participation processing. Step 2 defines the system input. Step 3 defines the system output. Step 4 receives the incoming participation request from the user. Step 5 updates the local event index for tracking the current participation event. Step 6 transforms the user request into a formal participation-event object so that it can be processed consistently by the platform. Step 7 determines the category of the generated participation event. Step 8—The correct processing handler is selected based on predefined routing rules.

Step 9 determines whether the occurrence pertains to authentication. Step 10 verifies the user credentials and checks the session authorization tokens. Step 11 checks if the authentication has failed. Step 12 generates a response indicating an authentication failure if invalid credentials or illegal access attempts are detected. Step 13 promptly transmits the failure response to avert illegal involvement. Step 14 closes the authentication-validation condition. Step 15 checks whether the event belongs to the verification category. Step 16 executes biometric identity verification and liveness analysis. Step 17 checks whether the verification process fails. Step 18 flags the participation event as suspicious activity if the biometric verification process is unsuccessful. Step 19 generates a verification-failure response. Step 20 returns the failure response to terminate suspicious activity processing. Step 21 closes the verification-validation condition. Step 22 checks whether the event belongs to the participation-processing category. Step 23 validates the requested participation operation (e.g., voting, commenting, registration, proposal submission, or rating activities). Step 24 checks if the user has sufficient participation eligibility and access permissions. Step 25 checks if the participation validation fails. Step 26 generates a participation-validation failure response when invalid or unauthorized participation requests are detected. Step 27 returns the validation-failure response. Step 28 closes the participation-validation condition. Step 29 checks whether the event belongs to the content-processing category. Step 30 normalizes and sanitizes the submitted participation content to remove invalid or malicious data. Step 31 forwards the processed content to the moderation and filtering module. Step 32 checks whether the moderation validation fails. Step 33 rejects the submitted content when moderation policies are violated. Step 34 generates a moderation-failure response. Step 35 returns the moderation response. Step 36 closes the content-validation condition. Step 37 checks whether the event belongs to the fraud detection category. Step 38 triggers anomaly-detection and enhanced security-verification procedures. Step 39 notifies the administrative monitoring module regarding suspicious platform activities. Step 40 generates a suspicious-activity response. Step 41 returns the suspicious-activity response to terminate further processing. Step 42 handles unsupported or undefined event categories. Step 43 generates an unsupported event response. Step 44 returns the unsupported event response. Step 45 closes the entire conditional event-processing structure. Step 46 executes the selected processing handler for successfully validated participation events. Step 47 updates the current participation status to reflect the latest user interaction and participation status within the platform. Step 48 stores structured participation records into the structured storage service for tracking and retrieval. Step 49 stores multimedia objects, uploaded documents, and audit logs in the unstructured storage service. Step 50 generates analytical records derived from the processed participation event and the updated participation state. Step 51 forwards the generated analytics information to the streaming analytics pipeline for real-time monitoring and analysis. Step 52 generates the final processing response to the user. Step 53 returns the final response, updated participation state, and analytical records as the result of the workflow.

### 4.1 Analysis of existing digital civic participation platforms

The study initiated with an exhaustive evaluation of digital civic participation platforms spanning both national and international borders. Global systems such as Decidim (Barcelona), Better Reykjavik, and Consul (Madrid) were evaluated for their pervasive implementation in participatory democracy and municipal administration. These platforms enable citizens to engage in online consultations, provide recommendations, vote on public initiatives, and discuss policy issues through digital interfaces. Kazakhstan's digital governance systems, including OpenAlmaty, e-Otinish, and e-Petition, were examined in conjunction with these global platforms. These are the main internet venues for citizen-government communication and public service feedback in the country. This analytical step identified architectural and operational restrictions that affect civic engagement infrastructure scalability, performance, and dependability. The technical analysis included identity verification, data processing, system architecture, analytics, and AI. The analysis found that many contemporary systems use monolithic or conventional client-server designs with user interface, data administration, and application logic. Comparable designs operate well under moderate workloads, but they often fail to scale during extended public consultations or election-related events. The computational efficacy of conventional systems can be approached through classical queueing theory. If the average arrival rate of participation requests is represented as λ and the processing capacity of the server is represented as μ, the expected system response time *T*can be expressed as in Equation 25:


$$\begin{array}{l} T\left(\lambda ,\mu \right)=\frac{1}{\mu -\lambda }=\frac{1}{\mu \left(1-ỹ\right)},ỹ=\frac{\lambda }{\mu },0\leq y<1 \end{array}$$ (25)

Where ỹ denotes system utilization ratio.

User inquiries approach system processing capacity, increasing response time rapidly. In civic participation settings with thousands of users, such factors often cause system degradation, service outages, or latency. The investigation also found that real-time analytics and AI are difficult to integrate into traditional infrastructures. Numerous platforms batch-process and analyze participation data. This strategy is useful for retrospective insights but not real-time decision-making or adaptive interface behavior. Campaign managers cannot rapidly spot unusual voting or manipulation. Citizen interactions can be formalized as a continuous event stream to better model involvement occurrences. The definition of a participation event sequence *E* is defined in Equation 26:


$$ℰ=\{e_{k}|e_{k}=(c_{i},t_{k},\tau_{k},d_{k}),\;c_{i}\in U,\;1\leq k\leq n$$ (26)

where *n* denotes the total number of participation events in the analyzed sequence.

This representation highlights the inherently event-driven nature of civic participation processes, in which each user interaction generates a discrete event that the system must process and analyze in real time.

The study found that many digital participation platforms lack identity verification, making online voting and consultation dangerous. Poor authentication methods encourage duplication, bot activity, and coordinated manipulation. Modern participation infrastructures use biometric or behavioral authentication. A simple verification model can utilize a probability function to verify a user's identity which can be defined in Equation 27.


$$\begin{array}{l} P_{auth}\left(c_{i}\right)=f\left(I_{i},B_{i},H_{i}\right) \end{array}$$ (27)

where *P*_*auth*_(*c*_*i*_) denotes the authentication probability for the citizen *c*_*i*_, *I*_*i*_ denotes identity information, *B*_*i*_ denotes biometric features, and *H*_*i*_ denotes behavioral characteristics.

If the resulting probability value falls below a predefined threshold, the system may trigger additional verification procedures or flag the event as suspicious. Current platforms exhibit insufficient moderation and fraud detection capabilities. Most solutions necessitate human moderation or rule-based filtering, which is inadequate for civic engagement. The swift influx of numerous comments, recommendations, and votes renders manual moderation unfeasible. Ruling-based systems may overlook intricate manipulations such as coordinated voting campaigns or artificial spam generation. Contemporary intelligent systems identify aberrant behavior in participation data streams using anomaly detection methodologies. Compute anomaly scores for participation events utilizing statistical or machine learning techniques: Equation 28 computes the anomaly score (standardized deviation).


$$\begin{array}{l} Score\left(e_{k}\right)=\frac{|x_{k}-\mu_{x}|}{\sigma_{x}} \end{array}$$ (28)

where *x*_*k*_ denotes the observed feature value of the event *e*_*k*_, while μ_*x*_ and σ_*x*_ denote the historical mean and standard deviation of that feature, respectively.

The study highlighted data governance and regulatory compliance, especially in governments where digital sovereignty demands sensitive citizen data to remain in national infrastructure alongside technical problems. National regulations mandate hybrid data localization, although multinational open civic interaction platforms sometimes ignore it. Comparative studies find civic engagement platforms lacking scalability, adaptability, security, and regulatory compliance. Traditional systems struggle with unpredictable participation peaks, massive data streams, and clever real-time analysis. These findings recommended a new architectural paradigm to solve these issues. An event-driven serverless architecture with AI modules and hybrid data localization is presented in this study. The proposed system models citizen interactions as dynamic event streams and uses cloud-native computing technologies to create a scalable and intelligent infrastructure for large-scale digital civic involvement that meets national data protection standards.

#### 4.1.1 Policy implications and real-world governance scenarios

The proposed Secure Event-Driven Serverless Participation Architecture (SESPA) has important policy implications for governments wanting to upgrade digital civic engagement platforms while maintaining security, scalability, transparency, and compliance with national data governance rules. The architecture is not only a pure technical solution, but a practical governance framework, able to support large-scale citizen participation efforts in smart cities and digital democracy ecosystems. By integrating event-driven processing, serverless computing, artificial intelligence, biometric verification, and hybrid data localization, the framework can help public institutions solve operational challenges commonly observed in modern digital participation environments. A typical example is the Decidim platform of Barcelona, a widely adopted open-source platform for participatory budgeting, public consultation, and collaborative policymaking. In large-scale public consultations, thousands of citizens may submit proposals, comments, votes, and policy recommendations at the same time. Traditional centralized infrastructures are often subject to scalability issues and increasing response times under such workloads. The proposed SESPA architecture can scale computational resources dynamically through serverless event processing mechanisms, thus enabling elastic scaling during participation peaks. In addition, the incorporation of real-time analytics and anomaly detection modules may allow municipal authorities to track participation patterns, identify suspicious activities, and produce evidence-based policy insights. These features may enhance the reliability, transparency, and responsiveness of digital governance processes. Another practical use case is the Consul platform in Madrid that supports citizen petitions, participatory governance projects, and policy consultation mechanisms. Large-scale participation campaigns can generate large volumes of citizen interactions and create opportunities for coordinated manipulation attempts, automated participation activities, or fraudulent voting behavior. The proposed architecture utilizes AI-enabled anomaly detection techniques to analyze streams of participation events to detect abnormal patterns of behavior and suspicious activities, while biometric verification and identity validation modules can be used to enhance authentication procedures and mitigate risks of duplicate registrations or attempts of impersonation. Therefore, the proposed framework can contribute to enhancing public trust, participation integrity, and accountability within digital democratic processes. The policy implications of the proposed architecture are particularly important in jurisdictions with strict digital sovereignty and data protection regulations in place. Kazakhstan offers a relevant example based on platforms such as e-Otinish, OpenAlmaty, and e-Petition that facilitate communication between the citizens and government, requests for services, and the collection of policy feedback. National regulations require sensitive citizen data to be kept within the control of the sovereign and to allow for the delivery of digital services in an efficient manner. The hybrid data localization model embedded in SESPA directly addresses this requirement by separating the personal identification data from anonymized participation events. Sensitive data can be kept within the national infrastructure, while anonymized analytical data can be processed in scalable cloud analytics services. This dual domain processing model allows for regulatory compliance and supports sophisticated participation analytics and decision-support capabilities.

Moreover, the proposed architecture provides additional policy benefits for the government agencies implementing smart-city transformation initiatives. The event-driven architecture enables scalable operations and resource utilization during elections, public consultations, emergency response campaigns, and participatory budgeting initiatives. The use of artificial intelligence enables automated moderation, anomaly detection, sentiment analysis, and behavioral analytics thus alleviating the administrative burden and improving the quality of decision-making. In addition, the architecture enhances transparency and accountability with the generation of structured participation records and real-time analytical reports that can help policymakers in evaluating the degree of citizen engagement and public sentiment.

Governance-wise, SESPA can aid evidence-based policy making by translating large volumes of citizen interactions into actionable analytical insights. Policymakers can almost in real time track participation levels, spot emerging public concerns, gauge policy acceptance, and monitor community responses. As such, the proposed architecture acts not only as a scalable technological infrastructure but also as a strategic policy-enabling framework that can underpin transparent, secure, citizen-centric, and regulation-compliant digital governance ecosystems.

### 4.2 Citizen participation event model

The CPEM was created as the conceptual underpinning of the proposed architecture based on the constraints of existing digital civic participation technologies. CPEM treats every citizen-participation platform interaction as a separate event in a continuous digital participation stream. Instead of sequentially processing user actions within tightly coupled application components, the CPEM approach models civic participation as a dynamic sequence of discrete events that can be processed asynchronously and distributed across a scalable cloud infrastructure. This approach treats user interactions like enrolling on the platform, submitting proposals, voting, or commenting on an initiative as atomic units. These activities generate a real-time participation stream that shows system civic engagement evolving. This allows the platform to accommodate large-scale public engagement procedures, such as municipal consultations or policy discussions, with thousands of interactions.

This formal model allows the system to record participant actions and contextual data for analytics, security verification, and adaptive interface responses. All system events constitute a continuous involvement stream that evolves. Since events are processed separately, the system may dynamically scale processing resources to meet participation intensity changes. In civic participation situations where public interest in certain issues may create rapid activity surges, this trait is crucial. Platform user state modeling is another major feature of the CPEM methodology. Each citizen using the system has a participation status that reflects their civic engagement. Observers might become active participants who offer recommendations or vote in consultations. The system represents a user's state, and new events cause state changes. The basic state transition mechanism can be defined in Equation 29:


$$\begin{array}{l} \zeta_{t+1}=\Gamma_{s}\left(\zeta_{t},e_{k}\right) \end{array}$$ (29)

where ζ_*t*_ denotes the user participation state at time *t*, and Γ_*s*_ denotes the state-transition function.

The state transition models can represent user behavior consistently in the participation system. Voting events can change a user's status from “eligible voter” to “vote recorded,” and comment posts can trigger sentiment analysis or moderation. CPEM frames civic involvement as events and state transitions, laying the groundwork for event-driven digital participation platforms. This modeling method has several benefits. The system can process participation events asynchronously, allowing architectural components to respond without central coordination. Second, event stream structure integrates with streaming analytics tools to quickly find coordinated activity, trends, and issues. Finally, event-driven representation allows adaptive user interfaces for citizen engagement behavior modifications. Theory and architecture of the proposed digital civic engagement platform are based on the Citizen Participation Event Model. Scalable processing, real-time analytics, and intelligent system responses are possible by structuring citizen interactions into event streams and establishing state transition mechanisms. Modern smart-city participation systems must be quick, secure, and democratic for huge user populations. An extended adaptive state-transition model with AI and streaming-awareness is defined as follows in Equation 30:


$$\begin{array}{l} \zeta_{t+1}=\Gamma_{s}\left(\zeta_{t},e_{k},A,\Lambda \left(t\right)\right) \end{array}$$ (30)

### 4.3 Event classification

All civic engagement platform events are categorized into functional classes in the proposed framework to facilitate easier processing, faster routing, and more thorough analysis. Because distinct processing pipelines, security checks, and storage techniques are used for citizen interactions, event classification is required. When distributed architecture computing components are joined together, the system can deliver events to them dynamically. Classification facilitates the execution of operational systems and advanced analytics. The categories of events determine how the platform processes, validates, saves, and analyzes civic engagement acts. These are the system event categories. AuthEvent focuses on identity verification and access control. Creating tokens, confirming credentials, logging in, and registering for citizenship. Only authorized users are able to vote or submit proposals on the participation platform thanks to authentication events. The event processing pipeline includes secure session management, authentication, and identity verification.

Verification events include both biometric identification verification and liveness detection. Several things occur when the system determines whether a platform user is not an automated agent or an impostor. Technologies for liveness detection and facial recognition are employed during this event. Verification events are necessary to combat fraud in digital democracy. The primary methods that participants engage in civic life are demonstrated by the Participation Event. We can participate in online consultations, grade suggestions, comment on government actions, vote on public ideas, and propose new policies. The civic engagement platform's primary features are these events, along with the data used for governance analytics and decision-making. The processes of producing, approving, and disseminating digital content on a platform are known as content events. Comments, uploading supporting documentation, administrator moderation, and content acceptance are a few examples. Good and community-focused civic debate is promoted via content events. Systems generate FraudEvents when they detect unusual activity. Attempts to vote more than once, coordinated automated operations, or other forms of system manipulation could result in these outcomes. Behavioral analytics modules and anomaly detection algorithms detect fraud in participation data streams. Examine and monitor event signals. We can have customizable interfaces, real-time dashboards, and data on how users use our website. They monitor public opinion, civic engagement, and participation for policymakers and administrators. To formalize the classification process, each incoming event *e* is assigned to a specific category through a classification function defined as in Equation 31:


$$\begin{array}{l} \kappa \left(e_{k}\right)=\arg\underset{\eta \in K}{\max}P\left(\eta |e_{k}\right) \end{array}$$ (31)

where κ(*e*_*k*_) denotes the assigned category of event *e*_*k*_, K denotes the set of possible event categories, and η is a candidate event category.

The distributed architecture routes events to processing pipelines after classification. Event types are sent to authentication, biometric verification, participation processing, or analytics modules by predefined rules. The platform handles huge participation loads with this categorization and routing technique, ensuring scalability, security, and dependability. The proposed system classifies events to manage civic engagement activities independently while contributing to a single participation data stream. This method increases system adaptability, real-time analytics, and the platform's abnormality detection and adaptive user engagement.

### 4.4 Event-driven serverless architecture

We suggest the Amazon Web Services event-driven serverless civic interaction platform. This design makes it possible to process citizen contact events that can grow without adding too much operational overhead, are fault-tolerant, and are reliable. Serverless techniques, on the other hand, dynamically assign computing resources to interface events, unlike monolithic systems. It can handle huge changes in user activity during public consultations, voting campaigns, and emergency government alerts. The architecture's multi-layer distributed event processing pipeline transmits events made by citizens to security, processing, and analytics modules. People can ask questions online or on their phones. Amazon Route 53 spreads traffic across infrastructure nodes and finds domain names for these searches. All requests go through the AWS WAF. It uses security rules and anomaly detection to filter traffic and keep communication safe. AWS Amplify lets us create secure tokens, federate identities, and register users for authentication and management. On Amplify, only users who have logged in can vote, leave comments, and suggest policies. After the request has been verified, the backend processing layer gets it. Amazon API Gateway acts as a middleman between the user interface and the backend service for serverless computing services. API Gateway checks requests, limits the number of requests to an API, sends authentication tokens, and sends data securely. The main event management design of the system considers that all user input is synchronized. Amazon EventBridge is the principal event bus that sends participation events to processing services. The routing process can be formally described using a mapping between the event space and the set of processing handlers. Let *E* = {*e*_1_, *e*_2_, …, *e*_*n*_} denote the set of participation events generated by users, and let H = {*h*_1_, *h*_2_, …, *h*_*m*_} represent the set of available processing services within the architecture. The event routing mechanism can then be expressed as the optimization problem which is determined in Equation 32.


$$\begin{array}{l} \rho \left(e_{k}\right)=\arg\underset{h_{j}\in H}{\max}\left(w_{1}P\left(h_{j}|e_{k}\right)+w_{2}\frac{1}{l\left(h_{j}\right)}+w_{3}Comp\left(h_{j},e_{k}\right)\right) \end{array}$$ (32)

where Hdenotes available processing handlers, *h*_*j*_d denotes a candidate handler, ρ(*e*_*k*_) denotes the routing decision, ↕(*h*_*j*_) denotes handler load, and *Comp*(*h*_*j*_, *e*_*k*_) denotes handler-event compatibility.

The system dynamically distributes participation events across processing services to balance computational burdens with this routing design. AWS Lambda functions execute event business logic after routing. Serverless Lambda allocates computer resources depending on events. These services check user permissions, vote, verify eligibility, and communicate with system databases. The system only calls Lambda functions when needed, saving processing resources and speeding response times. We may determine how much computing power the participation platform needs by calculating the overall cost of processing all events in a given time period.

The stream of participation events E_*t*_ occurring within time window *t*, and total system workload *W*(*t*) can then be defined as follows in Equation 33:


$$\begin{array}{l} \;E_{t}=\left\{e_{k}\in E^{*}|t_{k}\in \left[t,t+\Delta t\right]\right\} \end{array}$$

$$\begin{array}{l} W\left(t\right)=\underset{e_{k}\in E_{t}}{\sum }\left[c_{proc}\left(e_{k}\right)+\rho_{v}V\left(e_{k}\right)+\rho_{s}S_{store}\left(e_{k}\right)\right] \end{array}$$ (33)

where *c*_*proc*_(*e*_*k*_) denotes event-processing cost, *S*_*store*_(*e*_*k*_) denotes storage overhead, and ρ_*v*_, ρ_*s*_ denote workload weighting coefficients.

There are two free storage systems for processed participation data. Amazon DynamoDB stores structured data like vote results, participation records, and user interactions using NoSQL, a high-performance distributed application database. DynamoDB horizontally scales database operations, so participation data is always available even during peak demand. Amazon S3 stores submitted papers, biometric verification pictures, and multimedia content cheaply and reliably. Real-time governance analytics are provided using Amazon QuickSight analytical visualization and Amazon Kinesis event streaming. Kinesis detects trends, citizen engagement, and anomalous activity patterns in real time using streaming participation data. QuickSight's interactive dashboards display processed statistics. Their advice on civic engagement is invaluable to administrators and lawmakers. Streaming transformation turns raw participation events into analytical metrics. The real-time analytical insight generation is determined as follows in Equation 34:


$$\begin{array}{l} R_{an}\left(t\right)=\int_{t_{0}}^{t}\Gamma_{an}\left(E_{\tau }\right)\cdot \psi \left(\tau \right)d\tau \end{array}$$ (34)

where, Γ_*an*_ denotes the analytics extraction function, and ψ(τ) denotes the temporal event-prioritization weighting function.

This design allows the system to collect participation statistics and take real-time governance measures, helping policymakers make sensible decisions. The event-driven serverless design, however, is stable and flexible enough to accommodate a lot of online civic activity. Distributed event routing, serverless computing, cloud-native storage systems, and real-time analytics services keep civic engagement procedures responsive, safe, and adaptable to changing participation patterns.

### 4.5 AI integration

AI modules were integrated into the proposed design to improve the reliability, safety, and data analysis capabilities of the digital platform for civic involvement. These components enable the system to independently monitor individuals' activities, identify anomalous behavior patterns, and verify their identities. The simultaneous use of the system by thousands of individuals, making manual identity verification impossible, is enabled by AI, which is essential for large-scale digital governance systems. The AI components of architecture are responsible for a variety of critical duties, such as identifying anomalies, analyzing behavior, and verifying biometric identification.

Within the current prototype evaluation, these biometric verification operations were tested exclusively using synthetic verification workflows, simulated facial input scenarios, and artificial biometric test cases rather than real citizen biometric repositories.

These attributes guarantee that civic engagement processes are consistently transparent, truthful, and challenging to alter. To autonomously verify individuals' identities and security at events where they are present, the system uses machine learning services. The biometric similarity score between the input facial sample ϕ and stored the biometric template *b*_*j*_ is determined in Equation 35:


$$\begin{array}{l} Sim\left(\varphi ,b_{j}\right)=\frac{\sum_{i=1}^{n}w_{i}\psi_{i}\left(\varphi ,b_{j}\right)}{\sqrt{\sum_{i=1}^{n}w_{i}^{2}}} \end{array}$$ (35)

where, ψ_*i*_ denotes the *i*-th facial feature similarity function, and *n*denotes the number of extracted facial descriptors.

The system deems the face-matching stage successful and advances to liveness verification if the similarity score surpasses a predetermined identity verification threshold. The second stage assesses whether the image submitted is representative of a living human subject. The liveness detection function evaluates numerous visual and behavioral indicators to determine the likelihood that an input image corresponds to a live subject. This probability can be calculated in Equation 36:


$$\begin{array}{l} P_{live}\left(\varphi \right)=\sigma \left(\overset{m}{\underset{k=1}{\sum }}\alpha_{k}f_{k}\left(\varphi \right)+\overset{s}{\underset{q=1}{\sum }}\beta_{q}h_{q}\left(\varphi \right)+\gamma \right) \end{array}$$ (36)

where *P*_*live*_(φ) denotes the liveness probability of the facial sample φ, *f*_*k*_(φ) denotes visual liveness features, *h*_*q*_(φ) denotes behavioral liveness indicators, and γ is the bias calibration parameter.

By incorporating liveness detection and biometric verification into the event-driven architecture, the likelihood of automated manipulation and identity faking is significantly reduced. These AI systems only allow legal citizens to vote, discuss topics, and make suggestions. By incorporating machine learning verification into the distributed event pipeline, security can be checked in real time without hindering users' ability to interact with one another. The AI-powered biometric verification module enhances the reliability of digital civic participation systems by ensuring that the platform adheres to rigorous standards of security, authenticity, and dependability across a range of citizen engagement activities.

### 4.6 Anomaly detection

To identify patterns of dishonest behavior in civic participation processes, the system employs the Random Cut Forest (RCF) algorithm. Each event a person participates in is represented as a multidimensional feature vector encompassing the action category, temporal profile, contextual attributes, and device-level characteristics. The framework goes beyond a simple anomaly score; it computes an aggregated anomaly intensity across all trees in the forest, as shown in Equation 37. This approach accounts for partition density and structural changes within the forest, as well as deviations from expected local participation behavior. Finally, a robust adaptive thresholding mechanism, illustrated in Equation 38, uses the rolling distribution of anomaly intensities to determine whether an anomaly is suspicious. If the normalized anomaly value exceeds the critical boundary, the system flags the event as suspicious and initiates additional verification steps. Let the incoming participation observation be represented as a feature vector in a multidimensional behavioral space. The anomaly contribution of this observation across the forest can be modeled using Equation 37:


$$\begin{array}{l} ℜ\left(\xi_{s}\right)=\frac{1}{N_{f}}\overset{N_{f}}{\underset{f=1}{\sum }}\left[\frac{\sum_{\nu \in \Pi_{f}\left(\xi_{s}\right)}\alpha_{f,\nu }\log\left(1+\frac{∥\xi_{s}-c_{f,\nu }{∥}_{2}^{2}}{\varepsilon +\rho_{f,\nu }}\right)}{1+\sum_{\nu \in \Pi_{f}\left(\xi_{s}\right)}\alpha_{f,\nu }}\right] \end{array}$$ (37)

where ℜ(ξ_*s*_) denotes the aggregated anomaly intensity of participation observation ξ_*s*_, *N*_*f*_ denotes the total number of forest trees, Π_*f*_(ξ_*s*_) denotes traversed partitions within the tree *f*, α_*f*, ν_ denotes partition importance weights, and *c*_*f*, ν_ is the partition centroids

This formulation quantifies the deviation of the observation from anticipated local participation behavior, while also considering structural variation within the forest. The system uses the historical score distribution to create a dynamically normalized risk boundary. It then compares the anomaly intensity to this boundary to see if the participation record should be marked as suspicious. The decision function can be expressed by Equation 38:


$$D\left(\xi_{s}\right)=\{\begin{array}{cc} 1, & \frac{ℜ\left(\xi_{s}\right)-\mathrm{med}\left(\Lambda_{1:K}\right)}{\delta +1.4826\cdot \mathrm{MAD}\left(\Lambda_{1:K}\right)}+\beta_{c}\Omega \left(\xi_{s}\right)>\Theta_{crit}\\ 0, & otherwise \end{array}$$ (38)

where Ω(ξ_*s*_) denotes the contextual anomaly penalty term, β_*c*_ denotes the contextual amplification coefficient, and Θ_*crit*_ denotes the anomaly decision threshold.

A decision value of 1 means the system marks the participation event as suspicious and initiates further verification processes, which may involve heightened identity checks or an administrative review.

### 4.7 Hybrid data localization model

A hybrid data localization architecture has been implemented in accordance with the national digital sovereignty statutes of the Republic of Kazakhstan. This architecture enables the implementation of cloud processing systems to facilitate scalable analytics, while simultaneously safeguarding sensitive citizen data within sovereign infrastructure. A hybrid architecture divides data into two domains based on its sensitivity to privacy and legal regulations. The civic participation platform collects information that is organized into two primary categories by the system. Personal data (PD) is a collection of information that can be used to identify an individual, such as biometric verification records, citizen identifiers, authentication credentials, and other sensitive information associated with user accounts. In the current study, these data structures were modeled conceptually and evaluated using synthetic prototype records generated for controlled cloud experimentation rather than actual citizen identity information.

These data elements must remain within the national infrastructure to comply with laws and regulations that are designed to protect citizens' data. Analytical data (AD) is a collection of anonymized or aggregated participation data that is employed for the purpose of statistical analysis, the development of machine learning models, and the monitoring of civic engagement. This collection of metrics includes event frequency statistics, interaction metrics, and participation patterns that are devoid of personally identifiable information. Personal data is transformed into analytical data through an anonymization pipeline that prioritizes privacy. The transformation process can be interpreted as a multi-stage anonymization operator that maintains the analytical structure while eliminating identifiable attributes. Draw the original sensitive record as a carefully organized attribute vector. The anonymized analytical representation is obtained through the transformation which is given in Equation 39.


$$\begin{array}{l} a_{r}=Z_{\lambda }\left(p_{r}\right)=Z\left(p_{r}\backslash I_{r}\right)+\overset{m_{a}}{\underset{j=1}{\sum }}\theta_{j}\psi_{j}\left(p_{r}\right) \end{array}$$ (39)

where *p*_*r*_ denotes the *r*-th sensitive personal record, *a*_*r*_ denotes its anonymized analytical representation, Z_λ_denotes the parameterized anonymization transformation, *I*_*r*_ denotes identifiable attributes removed from *p*_*r*_, ψ_*j*_ denotes feature-extraction functions, θ_*j*_ denotes anonymization weighting coefficients, and *m*_*a*_ denotes the number of anonymized analytical features.

After the anonymization process, the architecture sends data between the sovereign data infrastructure and the cloud-based analytics environment. Because of this, the hybrid model works as a dual-domain processing framework with a sovereign data domain and a cloud analytics domain. The limitations on data governance that control this separation can be put into a restricted optimization formulation which is given in in Equation 40.


$$\begin{array}{l} \min_{\Xi }[\int_{U}\kappa_{1}{ℛ}_{privacy}\left(\Xi \left(d\right)\right)+\kappa_{2}{ℛ}_{latency}\left(\Xi \left(d\right)\right)\\ \;+\kappa_{3}{ℛ}_{utility}\left(\Xi \left(d\right)\right)du] \end{array}$$ (40)

subject to $\Xi (u)\in \{\begin{array}{cc} D_{SOV}, & if\;\chi (u)=1\\ D_{CLD}, & if\;\chi (u)=0 \end{array}$

where **Ξ**(*d*) represents the data placement decision, R_*privacy*_ represents the privacy exposure risk function, R_*latency*_ represents the data processing latency cost, R_*utility*_ *r*epresents the analytical usefulness of the data, κ_1_, κ_2_, κ_3_ are weighting parameters balancing privacy protection, processing performance, and analytical value, respectively, D_*SOV*_ is the sovereign national infrastructure domain, D_*CLD*_ the cloud analytics processing domain, and χ(*u*)is the data classification function determining whether the record contains personal attributes?

The system employs a dual-loop data processing design as a result. While anonymized participation events are transmitted to dispersed cloud analytics services for real-time processing and display, sensitive personal data remains within the national infrastructure. The platform can completely comply with national data protection regulations while simultaneously enabling the scalability and analytics capabilities required for contemporary digital governance systems. The proposed architecture strikes the ideal mix between data sovereignty, privacy protection, and computing scalability by segregating identity-sensitive data from analytical participation streams. This is crucial for creating trustworthy digital civic engagement platforms in smart cities.

Algorithm 2 illustrates AI-based verification, fraud detection, and hybrid data localization. Step 1 initializes the variables, which include the user request, event stream, event class, routing rules, serverless handlers, structured storage, unstructured storage, the current participation state, and analytics metrics. Steps 2–3 show input and output. Step 4 compares the captured facial image with stored biometric templates to measure how closely the two match. Step 5 checks whether the facial similarity score is below the similarity threshold. Step 6 shows that if the similarity score is too low, the system marks the verification status as failed. Step 7 describes that a fraud event is generated because the biometric match was unsuccessful. Step 8 shows that if the similarity score is acceptable, the algorithm proceeds to the next verification stage. Step 9 calculates the probability that the captured face belongs to a live person rather than a photograph or replayed video. Step 10 marks the end of the conditional section associated with the facial similarity test. Step 11 shows that the facial verification stage is completed. Step 12 checks whether the liveness probability is below the predefined threshold. Step 13 shows whether the liveness probability is low; the system marks the verification status as suspicious. Step 14 describes that a fraud event is generated due to possible spoofing or impersonation. In step 15, if the liveness probability is acceptable, the system proceeds to verification. In step 16, the verification status is set to verified. Step 17 closes the verification decision branch. Step 18 shows that the liveness verification process ends. Step 19 constructs an activity feature vector using contextual information such as action type, timestamp, user context, and device profile. Step 20 calculates the anomaly score using a machine-learning classifier based on the activity feature vector. Step 21 checks whether the anomaly score exceeds the predefined anomaly threshold. Step 22 shows that if the anomaly score is too high, the system marks the event as a fraud attempt. Step 23 triggers enhanced verification procedures and may involve administrative review. Step 24 shows the process of transforming personal data into anonymized data so it can be analyzed without exposing sensitive identity information. Step 25 checks whether the data contains sensitive identity attributes. Step 26 shows whether identity-sensitive data is present; the data is stored in a secure national infrastructure. Step 27 describes whether sensitive data is not present; the algorithm proceeds to the cloud-processing option. Step 28 anonymizes the data is sent to the cloud analytics environment. Step 29 closes the conditional data-localization branch. In step 30, the data localization decision process is completed. Step 31 marks the end of the anomaly-detection branch. Step 32 returns the verification status, fraud event indicator, and the data localization decision.

Algorithm 2 **AVFDHDL: AI-based verification, fraud detection, and hybrid data localization.**

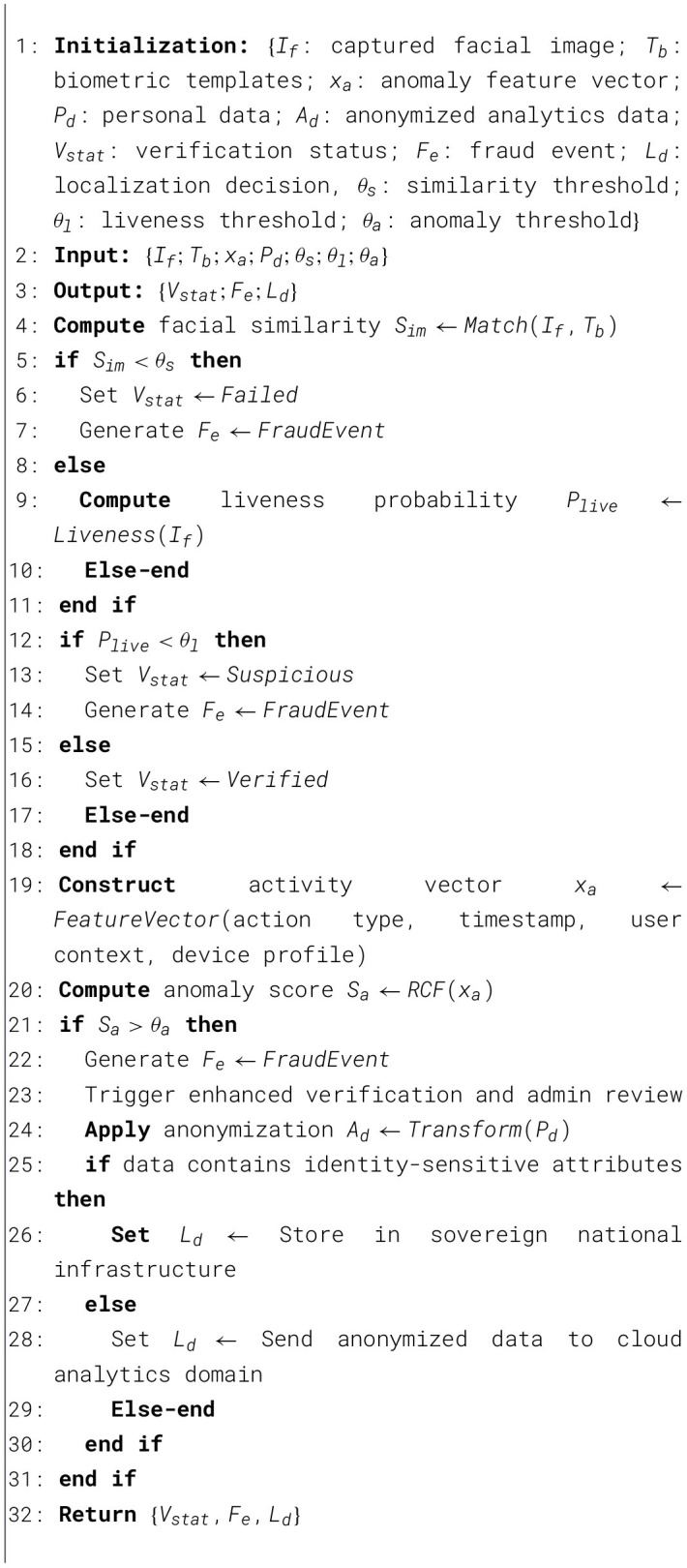

### 4.8 Security and privacy guarantees of the hybrid architecture

The hybrid data localization structure not only maintains private citizen data separate from analytical participation streams, but it also provides formal pledges about keeping data safe, processing it safely, and respecting people's privacy. For digital civic participation systems to work, users need to trust them, uphold the law, and restrict others from coming in without permission. The architecture contains a privacy-preserving perturbation technique that makes it impossible to get back to the original identification attributes from anonymized data streams. This keeps people's privacy safe while turning records of interactions between citizens into databases for analysis. In the realm of analysis, the anonymized record that comes from the transformation stage is called a structured vector representation. The privacy-preserving transformation can be determined as using Equation 41.


$$\begin{array}{l} {ã}_{s}=a_{s}+\overset{m_{a}}{\underset{j=1}{\sum }}\beta_{j}\varepsilon_{j},\varepsilon_{j}\sim N\left(0,\sigma_{j}^{2}\right) \end{array}$$ (41)

where ã_*s*_ denotes the privacy-preserved anonymized analytical record, *a*_*s*_ denotes the sanitized analytical representation before perturbation, ε_*j*_ denotes Gaussian perturbation noise, β_*j*_ denotes the perturbation weighting factor, and *m*_*a*_ denotes the number of anonymized analytical attributes.

This perturbation method keeps analytical aspects statistically helpful while making it impossible to put together personally identifying information. The system also protects access to participation data by using a layer that checks the data's integrity. There is a secure hash signature on every record of participation that is sent between parts of the system. This keeps the data the same and stops individuals from changing it without permission.

Let the secure integrity signature of records is defined in Equation 42.


$$\begin{array}{l} H_{s}=Hash\left(\overset{v}{\underset{r=1}{\sum }}\zeta_{r}\phi_{r}\left(\Upsilon^{\left(s\right)}\right)\;∥\;K_{\sec}\right) \end{array}$$ (42)

Where H_*s*_ represents the cryptographic integrity signature, ϕ_*r*_denotes feature extraction functions, ζ_*r*_represents weighting parameters applied to extracted features, *v* denotes the number of analytical feature transformations, K_sec_ denotes the secret cryptographic key, and ∥ is a concatenation of values.

This mechanism makes sure that any changes made to stored or sent data without permission can be found during verification processes. To further quantify the privacy protection properties of the architecture, the system evaluates the probability of identity inference from anonymized data streams. Let I be an adversarial inference function that tries to figure out identity attributes from records that have been anonymized. The privacy protection P_*risk*_ risk associated with a dataset of anonymized participation records can be modeled using Equation 43.


$$\begin{array}{l} P_{risk}=\frac{1}{N}\overset{N}{\underset{k=1}{\sum }}\mathbb{P}\left(I\left(\Upsilon^{\left(k\right)}\right)=\omega_{k}\right) \end{array}$$ (43)

Where *N* denotes the number of anonymized participation records, ℙ is the probability that the adversarial inference mechanism correctly reconstructs the identity information, **Υ**^(*k*)^ is the *k*-th anonymized analytical record, and ω_*k*_denotes the identity attribute.

Let Q(*t*) be the reliability function representing the probability that a participation event remains secure throughout the entire processing pipeline during the time interval *t*. This reliability of secure event processing over time interval *t* can be modeled by Equation 44.


$$\begin{array}{l} Q\left(t\right)=\exp\left(-\overset{t}{\underset{0}{\int }}\left[P_{1}\chi \left(\tau \right)+\rho_{2}\gamma \left(\tau \right)+\rho_{3}\delta \left(\tau \right)\right]d\tau \right) \end{array}$$ (44)

where χ(τ) is the potential risk, γ(τ)represents system vulnerability, δ(τ) rrepresents the probability of data integrity, and ϱ_1_, ϱ_2_, ϱ_3_represent the weighting coefficients associated with each security risk factor.

This formulation provides a probabilistic assessment of the efficiency of participation data within the distributed processing infrastructure. It indicates that the hybrid architecture does a good job of protecting privacy, data integrity, and safe processing. The proposed framework employs anonymization, perturbation mechanisms, cryptographic verification, and probabilistic security modeling to ensure that digital civic participation processes are secure, compliant with national legislation, and robust enough to mitigate potential security threats.

### 4.9 Digital service architecture

**Figure 2:**
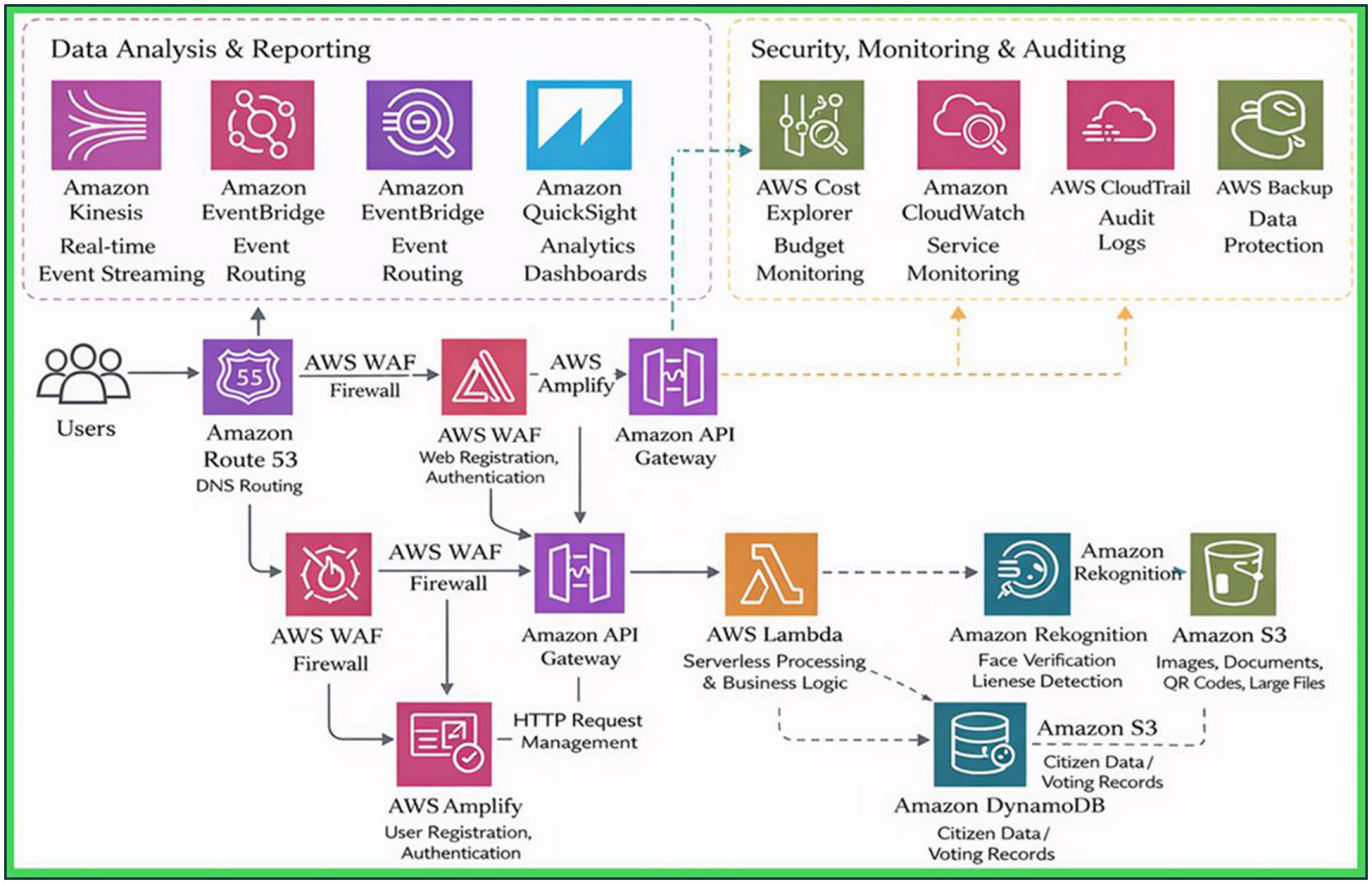
Event-driven serverless architecture for AI-enabled digital civic participation with biometric verification, streaming analytics, and hybrid data processing using AWS cloud services.

The architecture of the proposed digital service is depicted in Figure 2. It is a scalable, fault-tolerant, and intelligent system based on an event-driven approach and serverless computing. The key idea is that every user action is treated as an independent event, passing through a distributed processing flow. This ensures flexibility, the ability to instantly adapt resources to the current workload, integrate artificial intelligence modules into the computing circuit, and generate analytical signals in real time. The service begins with a user initiating an interaction through a mobile app or web interface. Each action generates an event, which is automatically routed to the event bus. Amazon EventBridge is used as the primary routing platform, allowing dynamic distribution of incoming events among the system's components. Each event, depending on its nature, whether it is an authentication action, biometric verification, survey participation, or a call to analytics components, is routed to the appropriate logic function implemented on AWS Lambda. This avoids the constant allocation of computing resources typical of traditional server systems and ensures automatic adaptation of the infrastructure to the current workload. A key feature of the proposed architecture is the integration of a biometric verification module. Using Amazon Rekognition, user images are compared with reference templates, ensuring high identification accuracy. This process is complemented by a built-in liveness detection model, which prevents attempts to spoof data by presenting photographs, videos, or other static media. The face comparison is determined in Equation 45.


$$\begin{array}{l} \text{match}\left(\varphi \right)=1\left[Sim\left(\varphi ,b_{j}\right)>\theta_{m}\right] \end{array}$$ (45)

Liveliness detection is calculated in Equation 46:


$$\begin{array}{l} P_{live}\left(\varphi \right)=P\left(real|\varphi \right) \end{array}$$ (46)

Equation 47 defines the fraud decision function, which determines whether an observed event φ should be classified as fraudulent based on behavioral matching and trust probability.


$$\text{aud}(\varphi )=\{\begin{array}{cc} 1, & match(\varphi )=0\;\vee \;P_{live}(\varphi )<\theta_{l}\\ 0, & otherwise \end{array}$$ (47)

Verification data undergoes multi-layered validation and is converted into events, which are then fed into the analytics module, where early signals are generated about potential risks and violations. Business logic processing, including voting management, comment processing, user permission assignment, and saving results, is handled by AWS Lambda functions interacting with data stores. Amazon DynamoDB is used to store structured information, enabling efficient query processing under high loads and ensuring low response times. Large data volumes, including images and log files, are stored in Amazon S3, ensuring long-term and cost-effective storage.

This approach creates a resilient data architecture in which structured and unstructured resources are processed optimally. The analytics layer plays a significant role in the overall architecture. Event streams generated during service operation are sent to Kinesis Data Streams, where they are sequentially analyzed. This layer is the key element of the system's intelligent behavior. It enables the real-time detection of anomalies in voting or user activity. Attention is paid to data localization and compliance with the legal requirements of the Republic of Kazakhstan. To this end, a dual-loop model has been developed, in which personal data is processed and stored exclusively in national data centers, while all non-identifiable events are transferred to the AWS cloud infrastructure for analytics and scalable processing. This ensures compliance with digital sovereignty requirements while preserving all the benefits of cloud technologies. This approach strikes a balance between security, performance, and ease of use. The dual-loop model is constructed as two linked domains. Sovereign contour and cloud contour. The sovereign contour is determined by Equation 48.


$$\begin{array}{l} \text{PD}=\left\{\text{id},\;\text{photo},\;\text{biometric}\right\} \end{array}$$ (48)

The data does not go outside the country. Analytical cloud contour is determined by Equation 49:


$$\begin{array}{l} \text{Anon}\left(\text{PD}\right)=\text{hash}\left(\text{PD}\right)+\text{minimal metadata} \end{array}$$ (49)

Formally, it is expressed in Equation 50:


$$\begin{array}{l} Data_{cloud}=Anon\left(PD\right)\cup E_{stream} \end{array}$$ (50)

The use of an event-driven architecture also ensures a high level of fault tolerance. Since each event is processed independently, local failures do not lead to a system shutdown. Components are automatically restarted if necessary, and event streams are rerouted, ensuring service continuity even under significant loads or temporary failure of individual components. An integral formal model of architecture is expressed in Equation 51:


$$\begin{array}{l} S=\langle E_{stream},Eb,\;H,Am,\;UI,\;Data_{hybrid}\rangle \end{array}$$ (51)

Where *E*_*stream*_ denotes data flow, *Eb* denotes EventBridge routing rules,), *Am* is analytical modules, *UI* is an adaptive interface, and *Data*_*hybrid*_ denotes a dual-loop data model.

## 5 Empirical testing and performance evaluation

To validate the proposed event-based architecture, a series of load and comparative tests were conducted to assess performance, scalability, resilience to peak loads, event processing costs, and the impact of AI modules on latency. The system was evaluated in the serverless AWS Lambda mode and as a containerized Kubernetes solution (EKS) under equivalent synthetic participation scenarios to analyze the architectural behavior, scalability characteristics, latency distribution and operational cost trends. All scenarios simulated real user activity related to city initiatives: mass voting, discussions, and application submissions. The profile was designed considering typical daily cycles of citizen activity.

### 5.1 Experimental methodology

The evaluation presented in this study was conducted using a working prototype implementation of SESPA deployed within an AWS cloud test environment. The experiments relied on controlled synthetic civic participation workloads generated using Apache JMeter and AWS Kinesis Data Generator to emulate realistic citizen interaction behavior under scalable participation scenarios. No live national deployment or operational governmental integration was performed during this study. For the experimental evaluation, we did not use any real citizen participation records, personally identifiable information, or operational governmental datasets. The proposed biometric verification, facial recognition, liveness detection, and anomaly detection modules were evaluated only on synthetic participation events, simulated verification workflows, artificial test inputs, and anonymized prototype scenarios created within the controlled AWS experimental environment. We did not collect, process, or store any human subjects, real biometric identities, or live citizen authentication records during this study.

We conducted a controlled cloud-based experiment to assess the efficacy, scalability, and stability of event-driven serverless architecture for digital civic participation platforms. The test checked how well the system worked, how quickly it replied, how it was set up without a server, and how many users could use it at once with varying workloads. The goal of these tests was to establish if the proposed architecture could accommodate a lot of people being involved in modern smart city government services with stable throughput and predictable latency. All the testing was done on an AWS test environment that was made to seem like a civic engagement system being used in the real world. An event streaming system turned on AWS Lambda functions that handled events. AWS Kinesis and API Gateway relayed participation events to the correct areas, while AWS CloudWatch watched over response times, invocation counts, error rates, and resource utilization. Serverless architecture is like a big platform for civic engagement where people may vote, remark, contribute, and ask questions utilizing digital tools. The Citizen Participation Event Model (CPEM) said that every action a user took constituted a participation event. This makes it possible for an event-driven pipeline to handle events without waiting for them to finish. Before saving or delivering analytics results to analytical components, serverless functions examined, analyzed, and calculated them. The logging and monitoring parts of the system recorded key performance indicators (KPIs) such as average latency, percentile latency distributions, system throughput, and event processing success rates so that the system could be properly tested under load.

#### 5.1.1 Cloud deployment configuration and reproducibility settings

The experimental prototype was deployed in the AWS us-east-1 (N. Virginia) region in a fully serverless cloud configuration. AWS Lambda functions were configured with 1,024 MB to 3,008 MB memory allocations, depending on the processing task. Authentication and participation processing functions used a 2,048 MB memory allocation with a timeout duration of 30 s. Analytics and anomaly-detection functions used 3,008 MB of memory allocation to support streaming computations and AI-based inference operations. Provisioned concurrency was enabled for critical participation-processing Lambda functions to minimize cold-start latency during high-load participation events. Amazon API Gateway was configured using REST API mode with request throttling enabled at 10,000 requests per second and burst limits of 5,000 concurrent requests. The payload size for simulated participation events ranged between 1 KB and 25 KB, depending on the event category, including voting requests, proposal submissions, comments, and simulated biometric verification metadata. Each experimental scenario generated between 100,000 and 1,200,000 participation events, depending on the concurrent-user configuration. Amazon DynamoDB was configured in on-demand capacity mode with automatic scaling enabled. Structured participation records, voting metadata, and user interaction logs were stored in DynamoDB tables with partition keys based on participation identifiers and timestamps. Unstructured participation data, including simulated biometric images, uploaded documents, audit logs, and participation artifacts, were stored in Amazon S3 Standard storage class. We set up AWS Kinesis Data Streams with 10 shards for event-stream ingestion and real-time analytics processing. Amazon EventBridge was the primary event-routing bus connecting API Gateway, Lambda services, analytic modules, and monitoring pipelines. For the experimental workflow, we used Apache JMeter and AWS Kinesis Data Generator to generate distributed synthetic civic participation workloads with randomized interarrival delays to simulate realistic citizen interaction behavior. Each experiment was executed five independent times under identical workload conditions, and the reported performance values correspond to the average measurements across all repetitions. The duration of individual experiments ranged from 15 to 45 min, depending on the workload intensity and concurrent-user profile. Concurrent-user workloads varied from 500 to 10,000 actively simulated participants. The cold-start was mitigated for critical Lambda functions with the use of provisioned concurrency and periodic warm-up invocation scheduling. AWS CloudWatch, AWS X-Ray, and CloudWatch Logs were used for system monitoring, latency tracing, invocation analysis, error tracking, and infrastructure-level observability for all experimental runs. The cost estimation is derived from the AWS pricing model for the duration of the experiment, encompassing Lambda invocation costs, provisioned concurrency charges, API Gateway request costs, DynamoDB on-demand pricing, Kinesis shard pricing, and S3 storage operations. The network transfer charges outside the AWS region were not included in the reported total cost of ownership calculations. Table 2 provides description of the used tools.

**Table 2:** Experimental cloud deployment and workload configuration.

Parameter	Configuration
Cloud region	AWS us-east-1 (N. Virginia)
Compute platform	AWS Lambda (Serverless)
Lambda memory allocation	1,024 MB−3,008 MB
Lambda timeout	30 s
Provisioned concurrency	Enabled
API gateway type	REST API
API gateway throttling	10,000 req/s
Burst limit	5,000 concurrent requests
DynamoDB mode	On-demand scaling
Event streaming platform	AWS Kinesis Data Streams
Number of kinesis shards	10
Object storage	Amazon S3 Standard
Monitoring tools	CloudWatch, AWS X-Ray
Load testing tools	Apache JMeter, Kinesis Data Generator
Payload size	1 KB−25 KB
Concurrent users	500–10,000
Number of repetitions	5
Experiment duration	15–45 min
Cold start handling	Provisioned concurrency + warm-up scheduling

#### 5.1.2 AI model training, validation, and experimental configuration

The artificial intelligence components integrated into the proposed SESPA prototype were evaluated using controlled synthetic participation datasets, simulated biometric verification workflows, and artificially generated participation behavior records specifically designed for experimental architectural validation. No real citizen biometric repositories, governmental identity databases, or personally identifiable citizen participation records were used during this study.

The experimental datasets were generated to emulate realistic large-scale civic participation scenarios, including voting activities, proposal submissions, participation comments, authentication attempts, and abnormal interaction behaviors. The synthetic participation dataset consisted of approximately 1.2 million generated participation events collected across multiple experimental workloads. The resulting dataset is comprised of real legitimate participation records and artificially injected anomalous activities such as duplicate participation attempts, automated bot activities, abnormal voting frequency, spoofed authentication attempts, and suspicious behavioral patterns. The biometric evaluation pipeline focused on architectural latency, verification orchestration, event-stream integration, and anomaly-response behavior, rather than production-grade facial recognition accuracy benchmarking against real biometric repositories. Simulated facial verification workflows and artificial facial embeddings were generated using randomized feature vectors to emulate legitimate and fraudulent authentication conditions for the evaluation of biometric verification. For anomaly detection, the component used the Random Cut Forest (RCF) algorithm with multidimensional vectors of behavioral features, including participation frequency, temporal interaction patterns, device profile indicators, geographical session variability, participation intervals, request entropy, and the measurement of authentication consistency. The RCF configuration used 100 trees, a shingle size of 8, a sample size of 512, and an anomaly scoring threshold dynamically adjusted using rolling median absolute deviation statistics.

A lightweight transformer-based Natural Language Processing model operated on synthetic civic feedback records that were synthesized from public-domain policy discussion templates and artificially generated citizen response patterns for the sentiment-analysis module. Text preprocessing involved token normalization, stop-word removal, punctuation filtering, and semantic embedding creation with pre-trained transformer embeddings. The synthetic datasets were split by a stratified 70/20/10 train-test-validate ratio to maintain the proportional distribution of normal and anomalous participation behaviors across the evaluation subsets. Further, each workload scenario was run five times repetitively to reduce stochastic variation and increase statistical confidence. Performance metrics included precision, recall, F1-score, verification latency, anomaly detection rate, false positive rate, and processing throughput. Confidence intervals were computed at the 95% confidence level using repeated experimental observations across independent workload executions. Average values reported in the experimental section, therefore, represent the mean values across repeated controlled experimental runs rather than single isolated executions. The primary objective of the AI evaluation was not to establish state-of-the-art machine learning superiority, but rather to validate the feasibility of integrating AI-assisted verification, anomaly detection, streaming analytics, and adaptive participation monitoring within a scalable event-driven serverless civic participation architecture.

#### 5.1.3 Baseline architecture comparison strategy

To evaluate the architectural characteristics of the proposed SESPA framework, a comparative experimental baseline was implemented using a containerized Kubernetes-based deployment on Amazon Elastic Kubernetes Service (EKS). The Kubernetes baseline was constructed specifically for controlled comparative evaluation purposes and was configured to execute equivalent civic participation workflows, including authentication requests, participation-event processing, event routing, storage operations, and analytics generation. Both the serverless SESPA implementation and the Kubernetes baseline processed the same synthetic participation workloads generated using the same event-generation pipeline and traffic profiles. The comparative analysis was mainly limited to architectural behavior, scalability response, latency distribution, infrastructure elasticity, and operational cost characteristics under similar participation scenarios, rather than claiming the universal superiority of one deployment paradigm over another. The Kubernetes implementation employed containerized microservices orchestrated through Kubernetes pods and horizontal pod autoscaling, whereas SESPA employed event-driven serverless orchestration using AWS Lambda and EventBridge services [Amazon Web Services (AWS), Inc. Seattle, Washington, USA]. Both environments were tested with similar participation workloads and the same application-level participation logic, but the results should be viewed as a prototype-level comparative architectural evaluation in a controlled cloud environment, not a definitive benchmark for all production-scale deployments. Differences in infrastructure tuning, cluster optimization policies, orchestration overhead, and cloud resource management strategies may affect the relative performance characteristics of alternative implementations. Therefore, the reported results show the suitability and operational advantages of the proposed event-driven serverless architecture for scalable digital civic participation workloads under the evaluated experimental conditions and not the absolute empirical superiority over all monolithic, microservice, or Kubernetes-based systems.

### 5.2 Latency: p50 /p75/p95/p99

**Figure 3:**
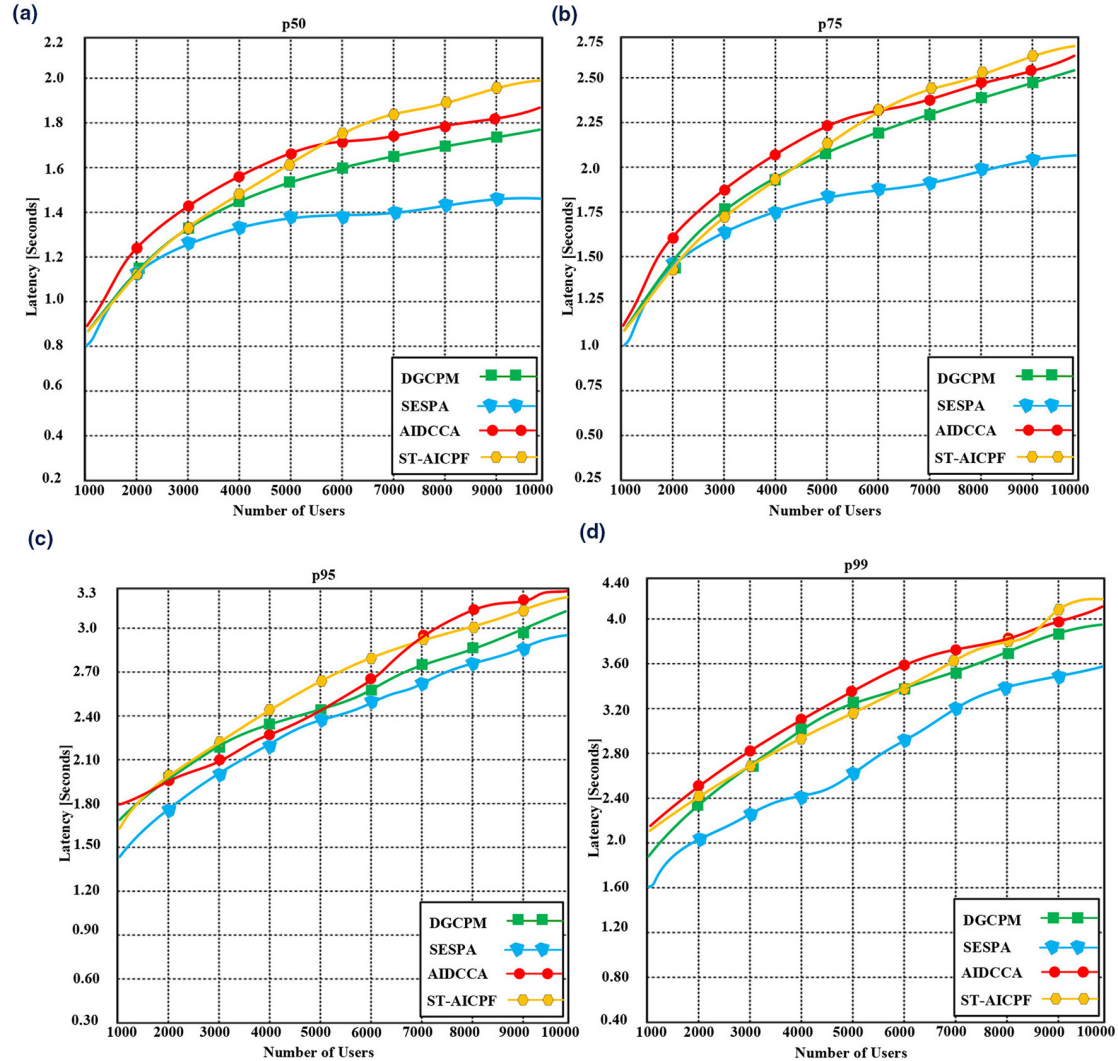
Comparative latency behavior of the proposed SESPA and competing civic participation architectures (ST-AICPF, AIDCCA, and DGCPM) under increasing concurrent user load: **(a)** p50 latency, **(b)** p75 latency, **(c)** p95 latency, and **(d)** p99 latency.

Latency (p50 /p75/p95/p99) is a way to measure how long it takes for a request to get a response in distributed systems, AI platforms, and cloud architectures. Different reaction time percentiles can be used to look at the average and worst-case delays. P50 latency is the middle latency for half of the system's requests. This shows what the average user experiences when things are normal. A low p50 latency means that requests are finished quickly. This number does not show performance issues that are slowing down requests. p75 latency is the time it takes for 75% of all system requests to be finished. Three-quarters of the requests are handled faster than this value, while the other 25% take longer. P95 is the latency for 95% of inquiries. Only 5% of slow inquiries go above the limit. This test illustrates how well the system works when there are a lot of users, when the processing task is very complicated, or when resources are limited for a short time. Finding p95 latency finds performance limits that median measurements don't catch. The p99 latency is higher than the slowest 1% of system queries. This number shows the worst-case scenario for system congestion, network delays, and heavy calculations. High p99 latency frequently means that the dependability or scalability of the distributed architecture is not good. The experiment is conducted to compare the proposed SESPA with competing architectures: Socio-technical AI civic participation framework (ST-AICPF) (Rossello M. et al., 2025), AI-driven civic consultation architecture (AIDCCA) (Dedema et al., 2025), Digital governance civic participation model (DGCPM) (Sadat et al., 2025). Figure 3a shows how the median response time of the architecture that was looked at changes as there is more work for the system to do. The median percentile (p50) is the longest time it takes to finish half of all service requests. When the system is working, this is what users normally see. We look at the proposed SESPA architecture next to three other frameworks: ST-AICPF, AIDCCA, and DGCPM. The results show that the SESPA design that was proposed always had the lowest median latency values in all of the tests. When there are about 1,000 inquiries, SESPA takes about 0.8 s to respond. It takes DGCPM and ST-AICPF roughly 0.85 to 0.90 s to respond, and AIDCCA takes about 0.92 s. This first difference shows how well the serverless event-driven execution strategy works to speed up response times and cut down on processing time, even when there isn't a lot of demand. The difference between SESPA and the other methods becomes clear as system activity rises. SESPA's latency stays at about 1.33 s, even when 4,000 operations are going on at the same time. The latency of DGCPM can be as high as 1.48 s, the latency of AIDCCA can be as high as 1.56 s, and the latency of ST-AICPF can be as high as 1.50 s. This design is around 10–15% better than the one that is most like it. It is simpler to tell how steady SESPA is when it needs to do more work. The latency for SESPA is 1.43 s, for DGCPM it is 1.70 s, for AIDCCA it is 1.78 s, and for ST-AICPF it is 1.88 s. The difference is between 20% and 30%. This suggests that the proposed architecture is preferable because it uses dynamic resource allocation and asynchronous event orchestration. The median response time for SESPA is about 1.47 s, even when the load is at its greatest. The time for DGCPM goes up to around 1.75 s, for AIDCCA it goes up to about 1.80 s, and for ST-AICPF it goes up to about 2.0 s. This illustrates that the approach has a far lower rate of latency rise than the other approaches. The median latency numbers demonstrate that SESPA is superior at meeting operational needs in general. The results show that the system works a lot better when we employ all three of these things at the same time: serverless computing, event-driven processing, and distributed cloud architecture.

Figure 3b shows the response time distribution for the 75th percentile. This is the latency threshold at which most requests are finished. This percentile illustrates how well the system works because it includes both normal operations and responses that are a little late when the load is high. At lower operating levels, the difference in performance between designs is still minor, but the SESPA method still works better. In about 1.0 s, SESPA responds to about 1,000 events. The latencies for DGCPM and ST-AICPF are between 1.10 and 1.12 s, whereas the latencies for AIDCCA are approximately 1.08 s. SESPA is still more efficient because it doesn't have to do as much orchestration. As the need for systems rises, the distinctions between designs become clear. When there are 3,000 operating events, SESPA keeps latency at roughly 1.65 s. AIDCCA goes up to about 1.88 s, ST-AICPF goes up to about 1.73 s, and DGCPM goes up to around 1.72 s. This means that the proposed architecture will make the reaction time about 8–12% shorter than the other options. The SESPA illustrates that with 5,000 queries, the latency is about 1.85 s. DGCPM and ST-AICPF take roughly 2.08 to 2.12 s, whereas AIDCCA takes about 2.22 s. As the workload increases, the difference between the two becomes clearer and clearer. This shows how useful it is to be able to easily add and remove resources in a serverless architecture. When there are a lot of operations (around 8,000), SESPA keeps the delay at about 2.00 s. The latency for DGCPM is 2.40 s, for AIDCCA it is 2.47 s, and for ST-AICPF it is 2.55 s. This is an improvement of about 20–25%, which means that SESPA can still do well even when it has more work to do. At the highest workload level, SESPA took roughly 2.07 s. DGCPM records around 2.55 s, AIDCCA records about 2.65 s, and ST-AICPF records about 2.75 s. The fact that the latency of the competing systems is growing slowly means that their scaling strategies aren't very good and that it takes longer to coordinate resources. These results suggest that SESPA can tolerate modest service delays well because it uses distributed event processing and can grow and shrink as needed. The architecture makes sure that most of the system's interactions are still fast. This is really vital for digital civic platforms, because users need to have fun and be actively involved.

Figure 3c shows the response time for the 95th percentile, which is the time it takes for the slowest 5% of requests to the system to respond. This number is commonly used to see how powerful a system is since it demonstrates how stable performance is when there is a lot of work to do, and resources are scarce. The SESPA architecture that is proposed is clearly better when the system is a little busy. According to SESPA, it takes about 1,000 operations to reply in about 1.45 s. The response time for DGCPM is about 1.60 s, for AIDCCA it is about 1.78 s, and for ST-AICPF it is about 1.70 s. This shows that SESPA cuts high-percentile latency by 10–20% compared to other choices. Even when the workload rises to roughly 3,000 events, SESPA retains a latency close to 2.00 s. But DGCPM goes up to around 2.20 s, AIDCCA goes up to about 2.10 s, and ST-AICPF goes up to about 2.25 s. The difference in speed is because SESPA uses asynchronous event pipelines, which keep processing queues from getting too full. SESPA records a delay of about 2.35 s after 5,000 encounters. DGCPM gets close to 2.45 s, AIDCCA gets close to 2.45–2.50 s, and ST-AICPF gets close to 2.65 s. The percentage difference right now is between 10% and 15%. This suggests that the proposed technique is more stable. Figure 3c shows the response time for the 95th percentile, which is the time it takes for the slowest 5% of requests to the system to respond. This number is commonly used to see how powerful a system is since it demonstrates how stable performance is when there is a lot of work to do, and resources are scarce. The proposed SESPA is clearly better when the system is a little busy. According to SESPA, it takes about 1,000 operations to reply in about 1.45 s. The response time for DGCPM is about 1.60 s, for AIDCCA it is about 1.78 s, and for ST-AICPF it is about 1.70 s. This shows that SESPA cuts high-percentile latency by 10–20% compared to other choices. Even when the workload rises to roughly 3,000 events, SESPA retains a latency close to 2.00 s. But DGCPM goes up to around 2.20 s, AIDCCA goes up to about 2.10 s, and ST-AICPF goes up to about 2.25 s. The difference in speed is because SESPA uses asynchronous event pipelines, which keep processing queues from getting too full. SESPA records a delay of about 2.35 s after 5,000 encounters. DGCPM gets close to 2.45 s, AIDCCA gets close to 2.45–2.50 s, and ST-AICPF gets close to 2.65 s. The percentage difference right now is between 10% and 15%. This suggests that the proposed technique is more stable. Figure 3d shows the 99th percentile latency, which is the longest response time for the slowest 1% of system operations. This is a very significant statistic for figuring out how reliable a system is since it demonstrates how terrible things may get for customers when there is a lot of traffic.

The SESPA indicates that when there are roughly 1,000 requests, the delay is about 1.6 s. DGCPM records for around 1.9 s, AIDCCA records for about 2.1 s, and ST-AICPF records for about 2.05 s. This initial difference demonstrates that SESPA is already more adept at managing conditions characterized by significant latency. When there are roughly 3,000 requests, SESPA keeps latency at about 2.25 s. DGCPM goes up to 2.75 s, AIDCCA goes up to 2.82 s, and ST-AICPF records about 2.70 s. This is around 15–20% better than the specified architecture. At 5,000 encounters, SESPA has a latency of roughly 2.60 s. This is smaller than the 3.25 s for DGCPM, the 3.35 s for AIDCCA, and the 3.20 s for ST-AICPF. The decline in severe latency data suggests that SESPA is working to make the system less limited. When there are roughly 8,000 interactions, SESPA retains latency at about 3.35 s. DGCPM goes up to 3.70 s, AIDCCA comes near to 3.85 s, and ST-AICPF records about 3.80 s. This means that the system is less likely to crash when things go wrong. When the workload is highest, SESPA has a latency of around 3.55 s, DGCPM has a latency of more than 3.95 s, AIDCCA has a latency of about 4.15 s, and ST-AICPF has a latency of more than 4.25 s. The results reveal that SESPA cuts the highest latency values by 15–25% compared to the other architectures. The results show that the proposed design can handle the longest reaction times, even when there is a lot of activity.

### 5.3 Relative cost per event

**Figure 4:**
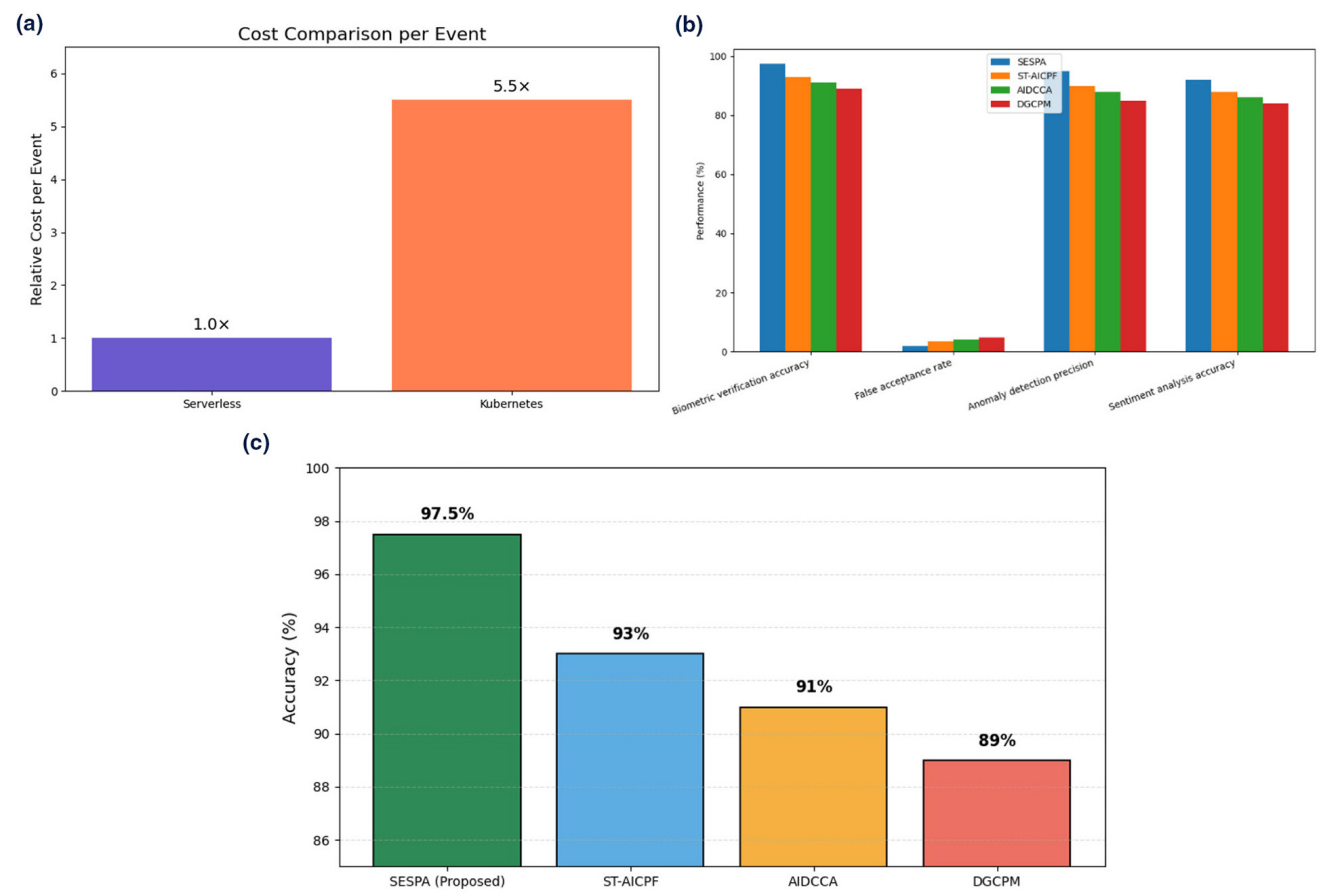
**(a)** Cost comparison per processed event between serverless and Kubernetes architectures. **(b)** Comparative performance evaluation of civic participation architectures. **(c)** Biometric verification accuracy comparison of civic participation architectures.

Serverless and containerized Kubernetes operational costs per processed event are shown in Figure 4a. People are testing the event-driven serverless system to see if it can handle a lot of digital civic involvement without costing too much. Compare event expenses starting with serverless. Designing without servers is cheap. A serverless technique with a basic cost of 1.0 × can process one civic engagement event. To process the same number of events, Kubernetes infrastructure costs 5.5 times more. Architecture distinguishes the two deployment models. Serverless architecture gives events instant computing power. Infrastructure resources are distributed during function execution. The usage of those resources determines expenses. This pay-per-execution mechanism prevents resource waste and adjusts running costs based on system usage. No matter how busy, Kubernetes must provide containers and cluster nodes with constant resources. When many people are undertaking civic activities and workloads vary, the cost difference is huge. Digital civic engagement platforms are busy during elections, public consultations, and policy discussions but not otherwise. Container architecture requires computing resources even when not in use, increasing costs. Serverless computing automatically adjusts resources to event streams. Thus, resources aren't wasted when demand is low. Serverless architecture becomes more valuable when it can handle more user events. The cost of designing a system with millions of participation events is different. Kubernetes-based deployments may cost 450% more than serverless solutions for the same task, with a cost ratio of 5.5 × . This disparity affects civic engagement platform TCO and is crucial for cities and governments seeking low-cost digital governance choices.

Serverless is cheaper because it doesn't manage infrastructure. Kubernetes clusters require extra nodes, container organization, and monitoring. Resources are needed, making things harder. Serverless solutions enable developers to focus on app logic and event handling without worrying about infrastructure. Growth is faster, inexpensive, and easier using SESPA. Event-driven serverless computing is affordable and suitable for large digital civic engagement platforms. This is especially true for smart city ecosystems that must remain profitable. Next-generation civic engagement infrastructures are cheaper with serverless designs. This allows governments to serve many people with fewer resources and infrastructure costs.

### 5.4 Security and analytics comparison of civic systems

Figure 4b compares the proposed SESPA with the other three competing architectures, ST-AICPF, AIDCCA, and DGCPM, based on several critical performance parameters for digital civic participation systems, including security verification, anomaly detection, and analytical intelligence. The metrics examined include the precision of biometric verification, the frequency of false acceptance, the reliability of anomaly detection, and the correctness of sentiment analysis. These things together show how smart and strong the platform is at keeping people safe when they engage with each other, and looking at data that has to do with policy. The SESPA architecture we presented surpassed nearly all the tests conducted, demonstrating superior performance compared to alternative solutions. This shows that it is a good idea to use an event-driven serverless architecture with AI-based verification and analytics modules. The proposed SESPA architecture has a biometric verification accuracy of about 97–98%. This is the best of all the systems that were tested. The ST-AICPF model is correct around 93% of the time, the AIDCCA model is correct about 91% of the time, and the DGCPM model is correct about 89% of the time. SESPA is more accurate since it incorporates advanced biometric validation algorithms in a pipeline for processing events in real time. This facilitates the prompt verification of individuals' identities during voting, commenting, or proposal submission. The false acceptance rate (FAR) is another important thing to think about. It tells us how likely it is that the system will let someone who shouldn't be there in. A lower FAR number means that security is better. The proposed SESPA design has the lowest false acceptance rate, which is about 2%. This means that it is a very safe way to verify someone's identity. On the other hand, the designs that compete with each other have larger FAR values: ST-AICPF is around 3%, AIDCCA is about 4%, and DGCPM is about 5%. The fact that SESPA has a lower false acceptance rate means that its AI-enhanced biometric verification system works and can prohibit people who should not be able to get in from getting in. The next number we looked at was the accuracy of anomaly detection. This shows us how well the system can find strange or suspicious behavior during civic participation events. SESPA can find weird items with about 95% accuracy. This is better than ST-AICPF (about 90%), AIDCCA (88%), and DGCPM (about 85%). This progress shows that the AI modules that come with the event-driven architecture work well. SESPA can find odd patterns more accurately by looking at streams of real-time participation data. This includes voting or participation events that seem odd. The last element in the photo is how well sentiment analysis works. This shows how well the computer can figure out what people are thinking and feeling based on what they say or how they discuss policies. The proposed architecture correctly classifies about 92% of the sentiments. ST-AICPF gets around 88%, AIDCCA gets about 86%, and DGCPM gets about 84%. This advancement shows that SESPA gives policymakers more accurate analytical insights by accurately understanding how people feel and how they are involved. Figure 4b shows that the SESPA architecture that is being supplied will improve security, dependability, and analytical intelligence all at the same time compared to the current civic participation frameworks. Using serverless computing, event-driven orchestration, and AI-based security modules all at the same time can make it easier to verify someone's identity, make authentication less likely to fail, find more anomalies, and get more accurate sentiment analysis. These results show that SESPA is a reliable and cutting-edge solution for the future generation of digital civic participation platforms. This is especially true in smart cities, where safe identity verification, reliable analytics, and infrastructure that can manage more citizen contact are very important.

### 5.5 Accuracy

Figure 4c demonstrates how well biometric verification works for SESPA, ST-AICPF, AIDCCA, and DGCPM. The accuracy of biometric authentication is an important way to determine how well digital civic participation systems work. SESPA has the greatest verification rate ever reported at 97.5%. This score shows that the method would be good at solving challenges with proving identities and showing participants correctly. AI-based biometric authentication systems make processing pipelines that are triggered by events more accurate. These modules are more reliable since they use adaptive authentication and real-time biometric signal identification verification. The ST-AICPF design's verification accuracy is 93%, which is 4.5 points lower than SESPA's. Even while ST-AICPF employs AI to look at civic action, it still doesn't do a good job of checking who people are. The technique of verifying makes it less accurate, which makes misclassification worse. AIDCCA's biometric verification is 91% accurate, which is 6.5 points lower than SESPA's. AIDCCA uses digital governance and public participation; its authentication modules may not be strong enough for biometric identity and adaptive verification systems. It could be hard to verify the System ID for whatever reason. DGCPM (89%) is the biometric verification that is the least accurate. If biometric and identity verification aren't employed, DGCPM could be 8.5 percentage points less accurate than SESPA.

The system may make more blunders when it has to verify more than one person. The results indicate how SESPA makes sure that biometric authentication works on the civic participation platform. AI-powered biometric verification technologies and event-driven processing that doesn't need a server make SESPA authentication faster and better. Operational monitoring makes event-driven processing work better and more accurately. Biometric authentication is preferable for digital governance with SESPA. Digital voting and public consultation systems need to be able to reliably validate users in order to make sure that people can vote and share their ideas online. Civic participation platforms are safer and more open when there are fewer authentication problems. They help people trust digital governance more. Figure 4c illustrates that SESPA identity verification is better than frameworks for civic involvement. The people in charge of a smart city and its enormous population use advanced digital civic engagement tools that are made better by SESPA's scalable, event-driven infrastructure and superior biometric verification algorithms.

### 5.6 Throughput

**Figure 5:**
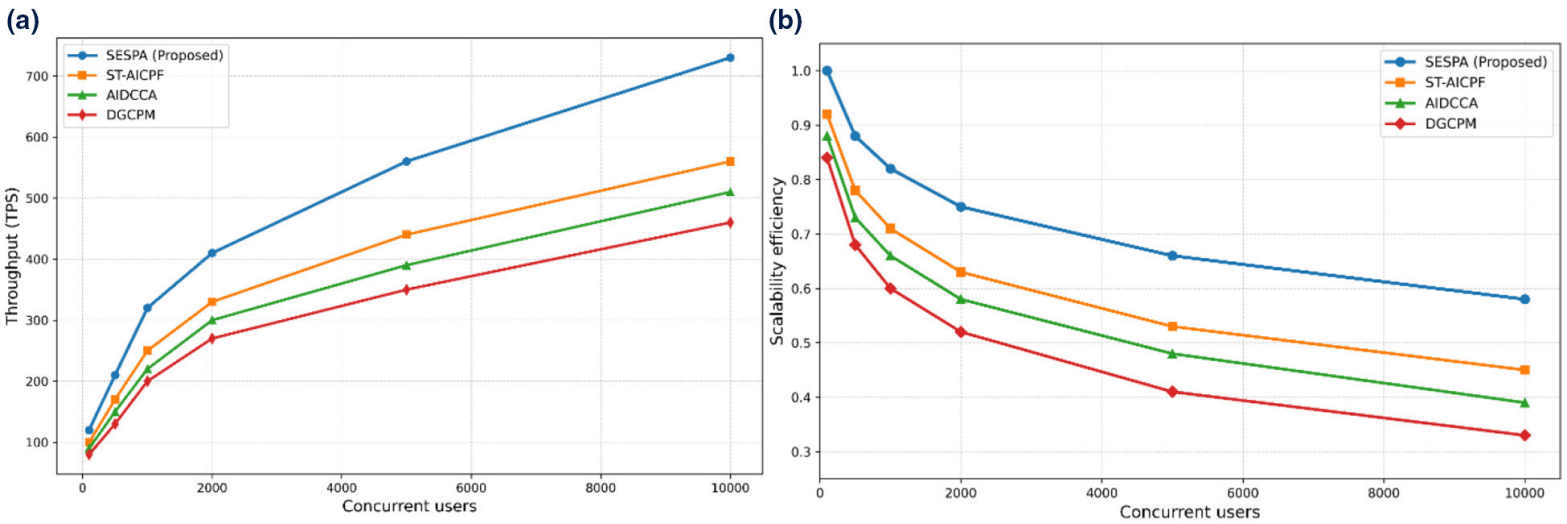
**(a)** Throughput comparison of SESPA and competing architectures under increasing system workload. **(b)** Scalability efficiency comparison of the proposed SESPA framework with existing approaches (ST-AICPF, AIDCCA, and DGCPM) under increasing concurrent user loads.

Figure 5a compares the throughput performance of four civic participation architectures: the proposed Secure Event-Driven Serverless Civic Participation Architecture (SESPA) and three existing frameworks: ST-AICPF, AIDCCA, and DGCPM. Throughput is the number of participation events the system can process per second. It is an important indicator of the scalability and processing capability of digital civic engagement platforms under increasing participation demand. The results demonstrate that the proposed SESPA architecture consistently achieves the highest throughput performance across all evaluated workload levels. The SESPA architecture processes approximately 120 transactions per second (TPS) at the lowest observed participation load. In comparison, ST-AICPF achieves roughly 100 TPS, AIDCCA records approximately 90 TPS, and DGCPM processes around 80 TPS. This initial difference indicates that SESPA already benefits from the efficiency of event-driven processing even when system demand is relatively moderate. As system workload increases, SESPA's throughput advantage becomes increasingly evident. At a participation level corresponding to roughly 500 concurrent operations, SESPA achieves about 210 TPS, whereas ST-AICPF processes approximately 170 TPS, AIDCCA about 150 TPS, and DGCPM roughly 130 TPS. This indicates that SESPA improves event processing capacity by approximately 20–30% compared with alternative architectures at this operational level.

When the workload reaches around 1,000 participation events, the proposed architecture continues to demonstrate superior scalability, achieving approximately 320 TPS. ST-AICPF processes about 250 TPS, AIDCCA approximately 220 TPS, and DGCPM around 200 TPS. The result reflects the efficiency of the serverless execution environment, which dynamically allocates computational resources. At higher demand levels, the scalability benefits of SESPA become even more pronounced. At approximately 2,000 concurrent participation events, SESPA achieves roughly 410 TPS, whereas ST-AICPF processes about 330 TPS, AIDCCA around 300 TPS, and DGCPM approximately 270 TPS. This improvement indicates the proposed architecture's ability to maintain efficient resource utilization and reduce processing bottlenecks as system demand grows. Under heavy operational conditions corresponding to approximately 5,000 participation events, SESPA processes about 560 TPS, while ST-AICPF achieves roughly 440 TPS, AIDCCA about 390 TPS, and DGCPM around 350 TPS. The gap between the proposed system and the competing architecture widens as system demand increases, indicating that SESPA scales more effectively under high workloads. At the highest evaluated participation level, approximately 10,000 concurrent events, SESPA reaches a throughput capacity of nearly 730 TPS, whereas ST-AICPF achieves about 560 TPS, AIDCCA around 510 TPS, and DGCPM approximately 460 TPS. The event-driven serverless approach can handle 30–40% more traffic than the other solutions. There are a few architectural reasons why SESPA has a higher throughput. First, the event-driven model allows civic involvement events to occur at different times so that people can participate in more than one activity. Second, serverless computing dynamically assigns computer resources based on demand. This reduces resource bottlenecks and improves workload distribution. A distributed cloud architecture allows the system to handle large amounts of data on how people interact with it without requiring additional infrastructure. Figure 5a illustrates that the SESPA design has a higher throughput than civic participation systems. Because it scales easily, the architecture is well-suited to large-scale digital governance settings such as smart cities and national civic engagement platforms, where thousands of people use the system simultaneously.

### 5.7 Scalability efficiency

Figure 5b indicates that the proposed SESPA is compared with the state-of-the-art methods AICPF, AIDCCA, and DGCPM. Scalability efficiency demonstrates how well a system can handle more work without losing processing power. It also shows how well computing resources are being used as more people use them. The results demonstrate that our proposed SESPA design always has the best scalability efficiency, no matter how much work there is to accomplish. At the first level of system demand, SESPA's scaling efficiency is about 1.00, which is the best it can be for this type of architecture. ST-AICPF records around 0.92, AIDCCA records about 0.88, and DGCPM records about 0.84, which is less than this. These early results show that the proposed architecture uses computing resources more efficiently, even when not many people are utilizing them. As the number of events that people can join increases, all architectures show a steady drop in scalability efficiency. When distributed digital governance systems are under a lot of stress, they have to deal with higher processing overhead, resource congestion, and network communication delays. This is why this behavior is predicted. But the SESPA design that has been proposed shows a far slower drop in efficiency than other choices. When there are about 1,000 events happening at the same time, the proposed architecture stays at an efficiency level of about 0.82. ST-AICPF drops to around 0.71, AIDCCA drops to about 0.66, and DGCPM drops to about 0.60, on the other side. Because of this disparity, SESPA can tolerate 15% to 25% more growth than the other designs can at this point in the system's operation. As more people want SESPA, its benefits become clearer. SESPA works around 0.75% of the time, or about 2,000 times. ST-AICPF works around 63% of the time, AIDCCA works about 58% of the time, and DGCPM works about 52% of the time. This means that the proposed design will use resources more efficiently and keep processing performance steadier as more individuals join. SESPA's efficiency stays at around 0.66 with about 5,000 occurrences. ST-AICPF's goes down to about 0.53, AIDCCA's to about 0.48, and DGCPM's to about 0.41. At this point, the contrast between the designs shows how well SESPA's event-driven serverless orchestration works to keep the system scalable, even when there is a lot of work to do. Even when the system is at its busiest, with about 10,000 events happening at the same time, the proposed architecture preserves an efficiency level above 0.58. On the other hand, ST-AICPF, AIDCCA, and DGCPM all drop to around 0.45, 0.39, and 0.33, respectively.

## 6 Discussion of results

Experiments show that SESPA is better than civic participation frameworks when it comes to performance, scalability, security, dependability, and cost-effectiveness. Studies show that digital consultation platforms and large civic engagement spaces in cities may hold up to 10,000 people at once. The results show that serverless architectures that are event-driven could transform how digital governance systems are built and used. The test was to evaluate if the method could fix the flaws with the civic engagement system. Several new users faced problems with early monolithic client-server systems. The more people use a system, the less responsive, scalable, and good at using its resources it gets. The answer is a design that includes AI modules, serverless computing, event-driven processing, and hybrid data localization. This connection makes the system perform better and more reliably in the real world when there are a lot of people using it. The speed at which the system replies influences how people feel about a digital civic participation platform. How rapid voting, policy, and government happen on the platform depends on how people respond. People may not use digital government services as much if they take a long time to respond.

The p50, p75, p95, and p99 latency percentiles were used to look at response time in the study. The data demonstrates how well the system performs; from the shortest time it takes to respond to the largest delay that could happen. The proposed design is faster than the competitors in every percentile. When there is a lot of stress, SESPA's average latency is 0.8 s, while the average latency of competitor systems is 0.9 s. Even though it only works a little better with smaller workloads, event-driven execution makes typical operation better. We get better at what we do when we do more of it. When the system is active with thousands of events happening at the same time, SESPA's delay keeps getting longer, but other systems respond more quickly. When there are more users, the method decreases the median time by 20–30% compared to current frameworks. This means that processing events at different times helps the system run more smoothly and enables people to get involved. Dissimilar latency percentiles are more dissimilar from each other. The p75 percentile shows that architecture can handle most interactions between systems. Even with a lot of attention, three-quarters of the queries are still answered in two s. Adding a delay to competing designs makes it harder for users to interact with them.

High-percentile delay measures (p95 and p99) are very important because they show how the system works when demand is at its peak. These numbers show us how busy resources are and how long it takes to do things. SESPA makes responsiveness and tail latency faster. This notion lowers high-percentile latency even when a lot of people are utilizing it, which is better than past civic engagement frameworks. There were a few architectural features that helped. In the beginning of event-driven processing, every interaction is an event. We can handle more than one interaction at once. Second, serverless computing gives us the computational power us need when we require it. As additional individuals join, the system automatically gives out computing resources to keep things running well. Third, a distributed cloud infrastructure gets rid of slowdowns in processing. The proposed architecture works according to latency. Digital civic participation systems need to let people engage with each other in real time and have pleased users for public involvement to work. Throughput, or the number of participation events that can be processed in a certain amount of time, is another important measure of how well a system works. Throughput shows how many users a platform can handle and how easy it is to grow. Compared to alternative architectures, the SESPA architecture frequently provides higher throughput. The suggested solution can handle more transactions per second than other options that do not require a lot of people to be involved. The more people who wish to take part, the bigger this benefit gets. SESPA can process thousands of events at once, hence it can manage more interactions per second than other approaches. When demand is at its maximum, the proposed system is 30–40% better than its competitors.

Serverless computing is superior most of the time since it can grow. Kubernetes persistent containers always have access to computational power, no matter what the workload is. Container orchestration systems can automatically add or remove resources; however, this involves management and slows down the distribution of resources.

While event-based serverless systems develop operations. People can do things on their own when they take part. This solution fixes issues with managing resources in containerized systems and lets the platform grow as more people use it. Event-driven design sends out events that engage the processing pipeline. Parallel execution speeds up processing and lets a lot of people use the service at the same time. We can use the design for platforms that let individuals from all over the country talk to each other and smart city systems that let thousands of people talk to each other. The experimental study looked at the cost and performance benefits of serverless architecture for civic engagement platforms. To work for big populations, governments need to pay for the costs of administering digital governance systems. This is less expensive than containerized Kubernetes because we do not need a server. Serverless architecture costs five times less per event than containerized architecture. The basics of serverless computing explain why there is a difference in price. Serverless systems only give functions the computing power they need while they are operating. Infrastructure resources that aren't used are not used. The pay-per-execution model establishes a balance between how much the system is used and how much it costs to run the firm. But containerized systems need both containers and cluster nodes. Even when only a few individuals are utilizing them, infrastructure pieces demand computing power. No matter how the technology is used, it costs a lot to run. When there are a lot of people voting or enquiring about policies, serverless solutions help civic engagement platforms. The plan lets governments reach a lot of people without needing to spend money on infrastructure.

We should consider how well the integrated AI module functions when we test it. Biometric verification, finding unusual patterns, and analyzing people's feelings all make the platform safer and better at analyzing data. The biometric verification accuracy of all the systems that were looked at was lower than what was expected. The AI-based verification pipeline for the event processing workflow makes this even better. It is difficult for people to misrepresent their identity or deploy bots when facial recognition, liveness detection, and behavioral analysis are used.

The proposed system has a lower rate of false acceptance than other systems. Digital governance security is better because people who aren't supposed to be able to utilize the participation platform are less likely to do so.

The anomaly detection module looks for strange patterns of participation in real time, which makes the system more trustworthy. Random Cut Forest can find rare votes, coordinated manipulation, and strange participation. Early identification of manipulation in the public decision-making system and civic involvement. Sentimental research helps policymakers understand what individuals are saying in comments. The system may uncover new public concerns and keep track of how people feel about them by going over the consultation papers. This skill helps policymakers make judgments that are based on facts. This study will change how digital governance infrastructure is built for future generations. We learned that event-driven serverless solutions make civic engagement platforms more scalable, efficient, and reliable. Technology handles a lot of participation data in real time by recognizing citizen interactions as events. With this infrastructure, governments may hold massive gatherings, digital voting, and policy discussions where everyone can speak their mind without any problems. AI modules improve the platform's analytics and security. Analytics modules demonstrate how individuals generally interact with each other, and AI-based verification makes sure that the processes for participation are reliable. Many places employ hybrid data localization to make it easier to follow the rules. Cloud infrastructure growth meets national data sovereignty rules by keeping personal information separate from anonymized analytical data. Table 3 provides a comprehensive performance of the proposed SESPA architecture against existing civic participation frameworks (ST-AICPF, AIDCCA, and DGCPM) across various evaluation metrics, including latency, throughput, scalability efficiency, biometric verification accuracy, anomaly detection capability, and cost efficiency.

**Table 3:** A comprehensive performance of the proposed SESPA architecture against existing civic participation frameworks (ST-AICPF, AIDCCA, and DGCPM) across various evaluation metrics, including latency, throughput, scalability efficiency, biometric verification accuracy, and anomaly detection capability, and cost efficiency.

System load/metric	Proposed SESPA	AIDCCA	ST-AICPF	DGCPM
Latency p50 (s)−1k load	0.80	0.92	0.90	0.85
Latency p50 (s)−4k load	1.33	1.56	1.50	1.48
Latency p50 (s)−8k load	1.43	1.78	1.88	1.70
Latency p50 (s)−10k load	1.47	1.80	2.00	1.75
Latency p75 (s)−1k load	1.00	1.08	1.12	1.10
Latency p75 (s)−3k load	1.65	1.88	1.73	1.72
Latency p75 (s)−5k load	1.85	2.22	2.12	2.08
Latency p75 (s)−8k load	2.00	2.47	2.55	2.40
Latency p75 (s)−10k load	2.07	2.65	2.75	2.55
Latency p95 (s)−1k load	1.45	1.78	1.70	1.60
Latency p95 (s)−3k load	2.00	2.10	2.25	2.20
Latency p95 (s)−5k load	2.35	2.50	2.65	2.45
Latency p95 (s)−8k load	2.75	3.15	3.00	2.90
Latency p95 (s)−10k load	2.95	3.30	3.20	3.10
Latency p99 (s)−1k load	1.60	2.10	2.05	1.90
Latency p99 (s)−3k load	2.25	2.82	2.70	2.75
Latency p99 (s)−5k load	2.60	3.35	3.20	3.25
Latency p99 (s)−8k load	3.35	3.85	3.80	3.70
Latency p99 (s)−10k load	3.55	4.15	4.25	3.95
Throughput (TPS)−100 load	120	90	100	80
Throughput (TPS)−500 load	210	150	170	130
Throughput (TPS)−1,000 load	320	220	250	200
Throughput (TPS)−2,000 load	410	300	330	270
Throughput (TPS)−5,000 load	560	390	440	350
Throughput (TPS)−10,000 load	730	510	560	460
Scalability efficiency—initial	1.00	0.88	0.92	0.84
Scalability efficiency−1,000 load	0.82	0.66	0.71	0.60
Scalability efficiency−2,000 load	0.75	0.58	0.63	0.52
Scalability efficiency−5,000 load	0.66	0.48	0.53	0.41
Scalability efficiency−1,0000 load	0.58	0.39	0.45	0.33
Biometric verification accuracy (%)	97.5	91	93	89
False acceptance rate (%)	2	4	3	5
Anomaly detection precision (%)	95	88	90	85
Sentiment analysis accuracy (%)	92	86	88	84
Relative cost per event	1.0 ×	–	–	–
Kubernetes deployment cost	–	–	5.5 ×	–

### 6.1 Limitations of proposed architecture

The proposed architecture has shown positive outcomes. However, there are a few drawbacks that need to be noted. The experimental evaluation was performed in a simulated cloud testing environment instead of a fully operational national civic participation platform. While the trials replicated authentic workloads and participation patterns, actual implementations may provide further complications associated with network variability, diverse user behaviors, and interaction with pre-existing government information systems. The proposed design is predominantly dependent on cloud infrastructure services. Although cloud platforms offer considerable scaling benefits, dependence on third-party infrastructure providers may lead to issues about long-term operational reliance, vendor lock-in, and regulatory limitations in specific regions. The efficacy of the artificial intelligence modules is contingent upon the quality of the training data and the precision of the machine learning models. Erroneous biometric recognition or anomaly detection algorithms may result in incorrect classifications. Consequently, ongoing model training and validation are essential to uphold dependable system performance. A further constraint pertains to the prerequisites for network connectivity. Digital civic engagement platforms depend on reliable internet connectivity for citizen interaction. In areas with inadequate digital infrastructure or unequal internet access, chances for participation may continue to be constrained despite enhancements in system scalability. Ultimately, while the suggested architecture exhibits significant performance enhancements relative to current frameworks, additional study is necessary to assess long-term operational stability and large-scale implementations involving millions of users.

The comparative assessment presented in this study is to be considered in the context of the controlled prototype implementation and experimental cloud environment. The Kubernetes-based baseline was implemented and tested under equivalent participation workloads; however, the performance characteristics of distributed cloud systems can vary based on infrastructure tuning policies, workload composition, orchestration strategies, autoscaling configurations, and deployment optimizations. Thus, the reported results should not be generalized as universal superiority claims for all serverless or containerized deployment scenarios. Future research may investigate the incorporation of supplementary technologies, such as blockchain-based audit trails, decentralized identity management systems, and edge computing infrastructures.

### 6.2 Policy implications and limitations

The proposed SESPA architecture carries important policy implications for digital governance, smart-city engagement and national data sovereignty. Real-world civic participation platforms such as Decidim, Consul, CitizenLab, OpenAlmaty, e-Otinish, and e-Petition demonstrate the growing need for scalable, transparent, and secure infrastructures supporting online consultations, public feedback, proposal submission, voting, and citizen-government communication. In such environments, the proposed event-driven serverless architecture can support large-scale participation processing, real-time monitoring of participation patterns, secure identity verification, automated anomaly detection, and evidence-based policy analytics. The hybrid data localization model is particularly relevant for countries with strict digital sovereignty requirements because sensitive personal data can remain within national infrastructure while anonymized participation events are processed through scalable cloud-based analytics.

From a policy perspective, architecture supports three important public-sector objectives. First, it supports civic inclusion by allowing large numbers of citizens to participate via digital platforms without overloading centralized infrastructures. Second, it improves trust in digital participation through identity verification, liveness detection, anomaly detection, and audit-oriented event processing. Third, it facilitates evidence-based governance by transforming citizen votes, comments, proposals, and participation records into actionable analytical reports for policymakers. For example, during city-level public consultations, the architecture can process thousands of simultaneous participation events, identify suspicious activities, generate participation summaries, and provide aggregated public sentiment indicators while preserving citizen privacy.

A limitation of the present study is that it does not involve human subjects. The proposed framework was evaluated as a controlled prototype using synthetic and workload-driven civic participation events designed to emulate realistic large-scale participation scenarios. No real citizen biometric records, personally identifiable information, public voting records, or human-subject responses were collected, stored, or analyzed. While this approach enabled systematic evaluation of the technical feasibility, scalability, security workflow, latency behavior, and architectural characteristics of the framework, it limits direct assessment of citizen acceptance, usability, trust, accessibility, demographic fairness, and behavioral diversity under real public participation conditions. The proposed method also has several practical limitations. This architecture is built on cloud native services and may be subject to vendor lock-in, regional service availability, volatility of cloud pricing, and platform-specific operational constraints. The use of AI-based biometric verification and anomaly detection mechanisms may also give rise to algorithmic bias, fairness concerns, false acceptance risk, and false rejection errors for demographic groups. The experimental evaluation with a synthetic workload may not fully capture the real-world civic engagement patterns, multilingual communication styles, political sensitivities, collusive manipulation attempts, or differential network access. The hybrid data localization model depends on strong legal governance, secure national infrastructure, inter-agency coordination, and continual compliance auditing. Serverless computing environments also suffer from cold-start latency, monitoring challenges, debugging difficulties, and dependence on resilient network connectivity. Automated verification and moderation mechanisms should be considered as supplements, rather than replacements, of human oversight, legal review, democratic accountability, and transparent public-sector decision-making. Future article comprises approval by institutional review boards, human-subject studies, usability assessment, fairness auditing of biometric and AI modules, privacy impact analysis, regulatory compliance evaluation, and pilot deployment with real civic participation stakeholders. Such investigations are needed in order to verify the effectiveness, reliability, and societal acceptability of the proposed architecture under real-world governance settings.

## 7 Conclusion and future work

This section concludes the entire article and provides the future directions.

### 7.1 Conclusion

This article empirically validates and proposes an event-driven, serverless architecture for a digital civic participation platform. The proposed SESPA is intended to function under conditions of strict reliability requirements, highly dynamic user activity, and adherence to digital sovereignty principles. In contrast to conventional monolithic and containerized solutions, the proposed approach perceives civic interaction as a continuous sequence of events, which facilitates the integration of AI and analytics modules, natural system scalability, and resilience to sudden load surges.

The formal CPEM was the primary architectural component of the study. This model delineates the actions of citizens as a series of events that are processed in a distributed environment. This formalization enabled the direct integration of biometric verification mechanisms into the system's event-driven framework, rather than their treatment as external add-ons. As a result, the platform is capable of not only ensuring the authenticity of participation and the sustainability of voting but also generating real-time analytics that support user interface adaptation and engagement monitoring. An empirical evaluation demonstrated stable system operation under loads of up to 10,000 concurrent users. Analysis of p50, p75, p95, and p99 latencies revealed predictable and controlled latency growth without performance degradation. The use of provisioned concurrency significantly reduced the impact of cold starts, making the architecture suitable for interactive mass participation scenarios. A comparison with a Kubernetes-based implementation revealed a significant advantage of the serverless approach in both scalability and total cost of ownership, which is especially important for municipal platforms with irregular activity cycles.

The scientific contributions of this work are as follows:

- A formal model for civic participation in events is developed for SESPA to represent civic engagement processes as event flows.
- A reference event-driven serverless architecture for digital participation with native integration of AI modules for verification is developed.
- A hybrid data localization model is proposed that ensures compliance with digital sovereignty requirements while preserving the benefits of cloud analytics.
- An empirical comparison of serverless and Kubernetes architectures in the context of e-participation platforms is conducted, with performance, scalability, and cost analyses.

The proposed hybrid data storage approach is particularly significant, since it involves processing personal information within a national network while anonymous events are handled in a cloud infrastructure. This method illustrates that contemporary cloud and AI technologies can be implemented in the public sector without contravening regulatory constraints and while preserving user trust. The findings of the study establish that event-driven serverless architecture serves as the technological basis for advanced digital civic participation platforms that are intelligent, adaptive, cost-efficient, and compliant with regulations. The proposed approach may serve as a reference framework for the creation of municipal and national e-participation services within smart city projects.

### 7.2 Future work

Future research will focus on enhancing the SESPA architecture by integrating blockchain-based transparency mechanisms to bolster trust and immutability in digital civic engagement processes. Subsequent study will examine the incorporation of federated learning models to enable collaborative AI training across multiple municipalities while ensuring the protection of citizen data privacy. Furthermore, research will evaluate the platform in national participation contexts with over 100,000 concurrent users and investigate advanced explainable AI models to improve transparency in anomaly detection. Ultimately, cross-country deployment studies will be conducted to evaluate the architecture's adaptability under diverse regulatory and digital sovereignty frameworks.

## Data Availability

The original contributions presented in the study are included in the article/supplementary material, further inquiries can be directed to the corresponding authors.
